# A model of functional thyroid disease status over the lifetime

**DOI:** 10.1371/journal.pone.0219769

**Published:** 2019-07-18

**Authors:** Michael W. Dzierlenga, Bruce C. Allen, Peyton L. Ward, Harvey J. Clewell, Matthew P. Longnecker

**Affiliations:** 1 ScitoVation, Research Triangle Park, North Carolina, United States of America; 2 Independent Consultant, Chapel Hill, North Carolina, United States of America; 3 Ramboll, Research Triangle Park, North Carolina, United States of America; North Carolina State University, UNITED STATES

## Abstract

Mathematical models of the natural history of disease can predict incidence rates based on prevalence data and support simulations of populations where thyroid function affects other aspects of physiology. We developed a Markov chain model of functional thyroid disease status over the lifetime. Subjects were in one of seven thyroid disease states at any given point in their lives [normal, subclinical hypothyroidism, overt hypothyroidism, treated thyroid disease (ever), subclinical hyperthyroidism, overt hyperthyroidism, and reverted to normal thyroid status]. We used a Bayesian approach to fitting model parameters. *A priori* probabilities of changing from each disease state to another per unit time were based on published data and summarized using meta-analysis, when possible. The probabilities of changing state were fitted to observed prevalence data based on the National Health and Nutrition Examination Survey 2007–2012. The fitted model provided a satisfactory fit to the observed prevalence data for each disease state, by sex and decade of age. For example, for males 50–59 years old, the observed prevalence of ever having treated thyroid disease was 4.4% and the predicted value was 4.6%. Comparing the incidence rates of treated disease predicted from our model with published values revealed that 82% were within a 4-fold difference. The differences seemed to be systematic and were consistent with expectation based on national iodine intakes. The model provided new and comprehensive estimates of functional thyroid disease incidence rates for the U.S. Because the model provides a reasonable fit to national prevalence data and predicts thyroid disease status over the lifetime, it is suitable for simulating populations, thereby making possible quantitative bias analyses of selected epidemiologic data reporting an association of thyroid disease with serum concentrations of environmental contaminants.

## Introduction

Mathematical models of the natural history of disease have been developed for many conditions [1–6], though to our knowledge this has not been done for thyroid disorders. Such models, where individuals are categorized according to disease state, are distinct from models of thyroid physiology that reflect the negative feedback loop in the thyroid axis [7–9]. Life-course models of thyroid disease status suitable for simulating a population have several potential applications. We needed such a model as a component of simulations in which we were evaluating the effect of thyroid disease on the renal excretion of xenobiotic compounds. The development of the model required that we quantitate the probability of thyroid disease development, progression, and treatment. This model allowed us to estimate sex- and age- specific incidence rates of treated hypothyroidism and hyperthyroidism in the U.S., which we believe have not previously been quantitated. The results may be of use in patient management and health care resource allocation.

**Fig 1:**
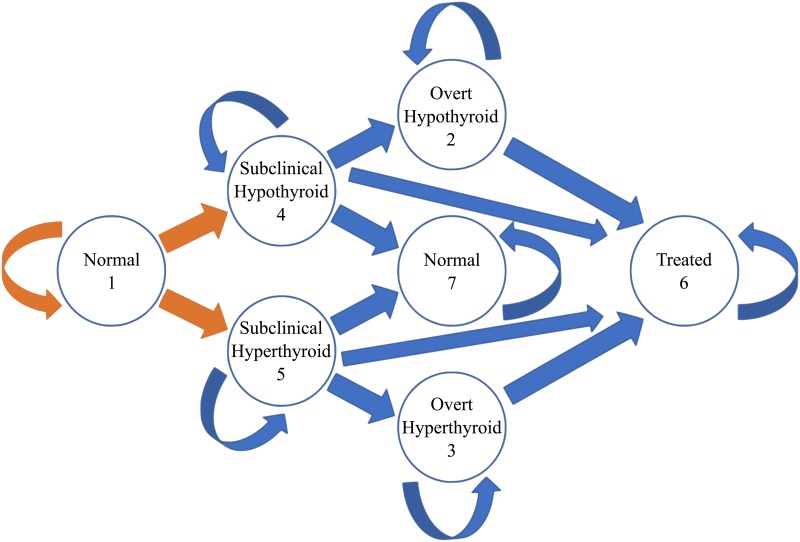
Diagram of the transitions in the Markov chain model. The transitions in this figure describe the possibility of remaining in the current state or changing to a state to the right, at each time increment. Each transition has a unique probability, with the initial development of subclinical disease also dependent on the sex and age of the subject.

Our mathematical method was to formulate the problem as a Markov chain model (MCM), a popular modeling approach that requires discretization of the model subject into distinct states, the transitions between which are described as functions of the current state, and not on the state history [10]. In our model, 7 states were used to describe thyroid disease status, as shown in Fig 1, with arrows representing possible transitions between states.

To estimate the probability of each transition in the MCM, we first summarized the published data on transition probabilities. These data were re-expressed as probability distributions and served as the *a priori* data. A Bayesian statistical approach updated the probability distributions so that they provided the best fit to U.S. national survey data on thyroid disease prevalence (target tables). The results were *a posteriori* probability distributions for the transitions that could realistically simulate thyroid disease status over the life-course of individuals in a population. The overall goals for our model were: 1) to realistically describe the probability of developing thyroid disease and its natural history for subjects over the course of their lives, and 2) on a population basis, create realistic estimates of thyroid disease prevalence, based on U.S. national survey data. The model will be useful for simulating the thyroid disease course for populations, thereby making possible quantitative bias analyses of selected epidemiologic data reporting an association of thyroid disease with serum concentrations of environmental contaminants.

## Materials and methods

In our model, six of the seven disease states were defined by the serum concentrations of thyroid stimulating hormone (TSH) and free thyroxine (fT4); people in the treated state were defined differently, as described below. Diseases of the thyroid that do not affect its function, such as most thyroid nodules, and iatrogenically-induced thyroid deficiency, such as treatment for cancer, were outside the scope of our model and would need to be described differently. Furthermore, the specific pathophysiology leading to a given thyroid state, e.g., subclinical hypothyroidism caused by Hashimoto’s disease, was not relevant to the needs of our model, as its focus was on the natural history of endocrinologic phenotype.

In the remainder of this section we: 1) describe the MCM and its underlying assumptions, 2) describe how we summarized the previously published data on transition probabilities, 3) describe how we calculated the prevalence of the target disease states, and then 4) explain the Bayesian approach to calibrating the transition probabilities that yielded our final life-course model of thyroid disease status.

### The Markov chain model (MCM)

**Fig 2:**
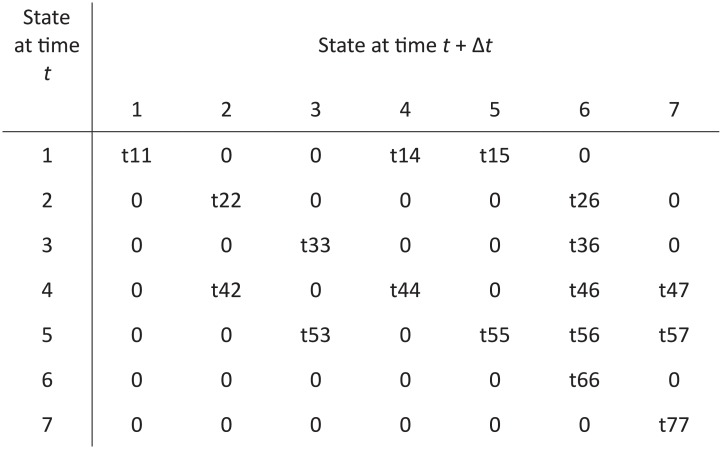
Transition matrix for the Markov chain model. Each entry in the matrix is the probability of a transition between disease states.

In the MCM, each arrow shown in Fig 1 has an associated probability of transition per time step. These probabilities can be depicted as a transition matrix (Fig 2). For example, if the time step were one year and t42 = 0.03, the annual probability of a subject with subclinical hypothyroidism becoming overtly hypothyroid (and untreated) is 0.03. A defining feature of a transition matrix for an MCM is that the probabilities across each row sum to one. The approach we used to develop the MCM required that we obtain estimates of the probability of transitioning from one thyroid disease state to another. For t14 and t15 we assumed the transition probabilities were sex- and age-specific because the incidence of treated hyperthyroidism and treated hypothyroidism vary by sex and age, implying that this is also the case for the incidence of subclinical disease. The age-specific transition probabilities were grouped into 10-year segments. Data were available to inform prior distributions for all age groups, but disease prevalence data, which were used to update the priors, were only available for ages 20–79. Because the incidences of hyperthyroidism and hypothyroidism below age 10 are essentially zero, we assumed everyone of that age was in the normal state. We did determine parameters for the 10–19 age group, but those transitions were only informed by their impact on the prevalences in the 20–29 age group. These choices allowed for a model which can be applied for the full lives of individuals from birth up to age 79, but with greater confidence in the adult ages (20–79).

### A priori transition probabilities

To identify publications on the probability of changing from one thyroid disease state to another, we searched PubMed in 2017 using terms applicable to each transition and limited the results to adults. For articles on the t14 and t15 transitions, we searched on “thyroid” and “incidence” and required that sex- and age-specific data on incidence rates be presented. To obtain information on t42, t46, and t47, we searched on “subclinical hypothyroidism” and “(course or natural history)” and did similar searches for t53, t56, and t57. For these transitions, we did not require age-specific data, primarily as a pragmatic measure, as it was clear that this level of detail was not available in the literature. The title and, if necessary, the abstract and full text of the resulting articles were reviewed for relevance. The results were supplemented by additional relevant articles that were in the bibliographies of the articles identified by the PubMed search.

Our search identified no studies that met our criteria for t14, t15, t46, and t56. Thus, alternative strategies were used to provide *a priori* parameter values for these. In a preliminary model that used *a priori* parameter values for the other parameters, setting t14 and t15 to three times the incidence rates of treated disease in Flynn et al. [11] resulted in a model that reproduced the rates of treated disease well. Thus, the sex- and 10-year age-group-specific rates of treated hypothyroidism and treated hyperthyroidism from Flynn et al. [11], multiplied by three, were used as *a priori* values for t14 and t15. For t46 and t56, we assigned an *a priori* annual transition probability of 0.02 because it seemed reasonable that they would be similar to the *a priori* values of t42 and t53 (see below). The *a priori* annual transition probabilities for t14, t15, t46, and t56 are presented in Table 1.

**Table 1:** *A priori* annual transition probabilities for development of subclinical thyroid disease by sex and age group (t14, t15, both derived from Flynn et al. [11]) and for changing from subclinical to treated thyroid disease (t46, and t56).

Transition Variable	Initial State	Next State	Sex	Age	Annual Transition Probability (%)
t14	Normal	Subclinical hypothyroidism	Male	10–19	0.027
				20–29	0.072
				30–39	0.129
				40–49	0.192
				50–59	0.357
				60–69	0.534
				70–79	0.807
t14	Normal	Subclinical hypothyroidism	Female	10–19	0.105
				20–29	0.549
				30–39	1.017
				40–49	1.821
				50–59	2.334
				60–69	2.718
				70–79	2.652
t15	Normal	Subclinical hyperthyroidism	Male	10–19	0.009
				20–29	0.018
				30–39	0.039
				40–49	0.063
				50–59	0.054
				60–69	0.081
				70–79	0.087
t15	Normal	Subclinical hyperthyroidism	Female	10–19	0.042
				20–29	0.234
				30–39	0.255
				40–49	0.270
				50–59	0.273
				60–69	0.336
				70–79	0.387
t46	Subclinical hypothyroidism	Treated	Both	all	2.00
t56	Subclinical hyperthyroidism	Treated	Both	all	2.00

For studies that were informative about t26, t36, t42, t47, t53, and t57, we abstracted the data on number of subjects at the beginning of follow-up, number of cases at the end of follow- up, and the years of follow-up (Table 2). For each study result, we calculated the proportion of subjects with the outcome of interest during follow-up (Pfu), and then identified the annual transition probability (pa) that would give the proportion with the outcome over the given period, using the formula ${\text{P}}_{\text{fu}}=\sum_{i=0}^{\text{n}-1}{\text{p}}_{\text{a}}*{\left(1-{\text{p}}_{\text{a}}\right)}^{\text{i}}$, where n = the number of years over which follow-up occurred. Years of follow-up were rounded to the nearest integer. The variance of pa was calculated as pa∙(1-pa)/N. We then calculated the weighted average (summary) pa for each transition, using inverse variance weights. It was not necessary to identify data on all transition probabilities in Fig 2, because with the requirement that row probabilities sum to 1, if all non-zero probabilities were known except one, the unknown value could be calculated by subtraction.

**Table 2:** Summary of data on annual transition probabilities (p) from studies of the natural history of thyroid disorders^a^.

Reference	Sex^b^	Number of subjects	Years of follow-up	Number of subjects who developed outcome	Proportion who developed outcome, P_fu_	Estimated p_a_	Standard deviation, p_a_	Weighted average p_a_
	Transition from subclinical hypothyroidism to overt hypothyroidism (t42)							
Díez 2004 [12]	b	107	2.6	28	0.262	0.095	0.028	
Huber 2002 [13]	f	82	9.2	23	0.280	0.036	0.021	
Imaizumi 2011 [14]	b	71	4.2	5	0.070	0.019	0.016	
Park 2013 [15]	b	169	5.0	19	0.112	0.024	0.012	
Rosário 2016 [16]^c^	f	252	5.0	48	0.190	0.042	0.013	
Somwaru 2012 [17]^d^	b	369	4.0	18	0.050	0.013	0.006	
Vanderpump 1995 [18]^e^	f	44	20.0	24	0.550	0.039	0.029	
								0.02
	Transition from subclinical hypothyroidism to normal thyroid state (t47)^f^							
Diez 2004 [12]	b	107	2.6	40	0.374	0.144	0.034	
Imaizumi 2011 [14]	b	71	4.2	38	0.535	0.174	0.045	
Park 2013 [15]	b	169	5.0	80	0.473	0.121	0.025	
Rosário 2016 [16]	f	252	5.0	57	0.228	0.050	0.014	
Somwaru 2012 [17]	b	369	4.0	92	0.250	0.070	0.013	
								0.08
	Transition from overt hypothyroidism to treated hypothyroidism (t26)							
Åsvold 2012 [19]^g^	b	418	11.1	412	0.990	0.370	0.024	
Vanderpump 1995 [18]^h^	b	79	20.0	51	0.646	0.050	0.025	
								0.21
	Transition from subclinical hyperthyroidism to overt hyperthyroidism (t53)							
Rosario 2010 [20]	f	102	3.4	3	0.029	0.010	0.010	
Vadiveloo 2011 [21]^i^	b	2024	7.0	494	0.244	0.039	0.004	
								0.03
	Transition from subclinical hyperthyroidism to normal thyroid status (t57)							
Rosario 2010 [20]	f	102	3.4	24	0.240	0.087	0.030	
Vadiveloo 2011 [21]	b	2024	7.0	721	0.360	0.062	0.005	
								0.06
	Transition from overt hyperthyroidism to treated hyperthyroidism (t36)							
Åsvold 2012 [19]^g^	b	120	11.1	112	0.930	0.230	0.038	
Vanderpump 1995 [18]^h^	b	20	20.0	15	0.759	0.067	0.056	
								0.16

^a^ Some values presented have been rounded to simplify the presentation.

^b^ “f” represents female subjects, “b” represent both male and female subjects.

^c^ The percent of subjects was based on those followed and applied to the n at baseline.

^d^ Somwaru et al. [17]’s results were presented as what occurred from years 0–2 of follow-up, and what occurred from years 2–4 of follow-up; the data we required for 0–4 years were not presented in the paper. We used the data presented for the two periods to estimate the proportion for the 0–4 period and used the n of subjects with observations at year 2 in the denominator.

^e^ We used Vanderpump et al. [18]’s Fig 2 to estimate that 5% of 875 subjects had increased TSH at baseline.

^f^ Results from Huber et al. 2002 were excluded from the summary because the pa value was an outlier compared with the others (0.004, SD 0.007, based on 4% of subjects reverting to normal), and because the initial subject group of 82 included patients with iatrogenic subclinical hypothyroidism.

^g^ The results shown for Åsvold et al. [19] were calculated from the data given on their page 95, Results, second paragraph.

^h^ The results shown for Vanderpump et al. [18] were calculated from the data given in their Table 4. For hypothyroidism, values for spontaneous disease were used.

^i^ We used the proportion of subjects among those followed to 7 years from their Table 3.

### Calculation of target tables of disease prevalence

**Fig 3:**
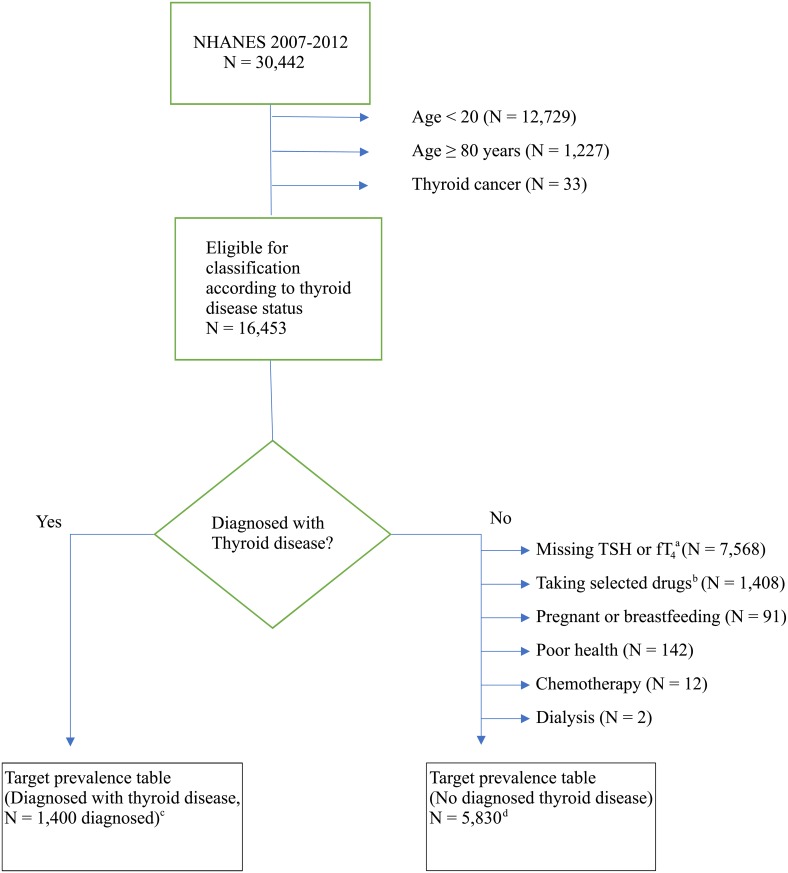
Flow chart showing population selection from the total NHANES participants to the target tables for the MCM. (a) For subjects in the 2007 to 2008 wave of NHANES, thyroid hormone levels were measured in all subjects over age 12 not meeting other exclusion criteria. For subjects from the 2009 to 2010 and 2011 to 2012 waves of NHANES, thyroid hormone levels were measured in a one third subsample of subjects over 12 not meeting other exclusion criteria. (b) Taking drugs that affect thyroid hormone concentrations. (c) Reported ever having thyroid disease, or on thyroid medication. Crude prevalence of ever diagnosed with thyroid disease was 8.5% (1,400/16,453). (d) These people were classified according to thyroid disease status on the basis of their serum concentrations of TSH and fT_4_.

We defined the prevalence of target disease states using data from the National Health and Nutrition Examination Survey (NHANES) [22]. Subjects aged < 20 years or ≥ 80 years or who reported ever having thyroid cancer were excluded (Fig 3). All analyses of the NHANES data incorporated the sampling parameters, to make the estimates representative of the U.S. population. Prevalence data for ages 10–19 were not used in the target tables because the NHANES did not ascertain thyroid disease status in this group.

The first set of target disease prevalences was lifetime prevalence of diagnosed thyroid disease. Individuals were considered to have thyroid disease if they reported ever being diagnosed with a thyroid condition or if they were taking one of several thyroid replacement or suppression drugs. The list of thyroid disease drugs is provided in the supplemental materials (Table A in S1 Supporting Information).

The second set of target disease prevalences comprised prevalences of undiagnosed thyroid diseases. For this analysis we excluded subjects who were missing serum concentration of TSH or fT4 or were taking drugs that interfere with thyroid hormone concentration. Furthermore, we excluded those who were pregnant or breastfeeding, described themselves as being in "poor health", were undergoing chemotherapy or were on dialysis, due to disruptions in thyroid hormone concentrations associated with those conditions. We reviewed 1) data on the normal reference range for TSH and fT4 for NHANES specifically [23], 2) hormone concentrations considered diagnostic by professional societies or experts [24–26], and 3) criteria from Hollowell et al. [27] for thyroid disease categories based on the NHANES III data, which used an older generation TSH assay. Among the reportedly healthy individuals we used the following diagnostic criteria for thyroid disease: overt hypothyroidism, TSH > 4 μIU/mL and fT4 < 0.5 ng/dL; overt hyperthyroidism, TSH < 0.2 μIU/mL and fT4 > 1.28 ng/dL; subclinical hypothyroidism, TSH > 4 μIU/mL and fT4 in the normal range; and subclinical hyperthyroidism, TSH < 0.2 μIU/mL and fT4 in the normal range. Variation in criteria for the definition of thyroid disease is well recognized [24–26]. The TSH > 4 μIU/mL criterion for hypothyroidism that we used is similar to the value of 4.12 μIU/mL recommended by professional society guidelines, and lower than the 4.5 μIU/mL used by Hollowell et al. [27]. The TSH < 0.2 μIU/mL criterion for hyperthyroidism that we used is higher than the value of 0.1 μIU/mL used by Hollowell and is lower than the value of 0.45 μIU/mL recommended by professional societies [26]. The population-based normal range for fT4 that we used, 0.5–1.28 ng/dL, was slightly different from the 0.61–1.12 ng/dL given in the NHANES laboratory documentation [28].

For both target tables, subjects were sorted by sex and ten-year age groups, starting from 20–29 and ending at 70–79. To calculate prevalence for a given age group, cases were divided by the population in their respective age group (Tables 3 and 4).

**Table 3:** Target and simulated sex- and age-specific prevalence of diagnosed thyroid disease^a^. The target (observed) values were from the NHANES, based on whether the participant reported ever being diagnosed with a thyroid condition or if they were taking one of several thyroid replacement or suppression drugs. See methods section (subheading “Calculation of target tables of disease prevalence”) and Fig 3 for details. The predicted values are of those ever having been treated for functional thyroid disease. See methods and discussion sections (subheading “Likelihood calculations” and “Predicted prevalences” respectively) for details.

Sex	Age Group (yr)	Observed (%)	Predicted (%)
Male	20–29	0.97	0.56
	30–39	1.56	1.71
	40–49	3.30	3.20
	50–59	4.42	4.62
	60–69	7.63	6.45
	70–79	8.19	8.84
	Mean^b^	4.10	3.93
Female	20–29	5.04	4.45
	30–39	7.28	8.33
	40–49	13.95	13.31
	50–59	18.07	17.66
	60–69	25.23	22.20
	70–79	28.22	25.48
	Mean^b^	15.41	14.48

^a^ The simulated prevalence was from the calculation of the prevalence in each sex and age group directly from the parameter set (no simulation needed).

^b^ Mean prevalence weighted by the number of observed individuals in each age group.

**Table 4:** Target and simulated sex-specific prevalence of undiagnosed thyroid disease^a^.

Sex	Age group (y)	Subclinical hypothyroidism		Overt hypothyroidsim		Subclinical hyperthyroidism		Overt hyperthyroidism	
		Observed (%)	Predicted (%)	Observed (%)	Predicted (%)	Observed (%)	Predicted (%)	Observed (%)	Predicted (%)
Male	20–29	3.26	1.43	0.15	0.03	0.15	0.10	0.15	0.03
	30–39	3.85	2.53	0.15	0.07	0.44	0.16	0.00	0.04
	40–49	3.45	2.51	0.00	0.08	0.16	0.14	0.00	0.04
	50–59	4.43	2.94	0.00	0.09	0.00	0.16	0.00	0.04
	60–69	5.53	4.19	0.00	0.12	0.22	0.22	0.22	0.06
	70–79	6.28	5.09	0.00	0.15	0.00	0.27	0.00	0.08
	Mean^b^	4.18	2.80	0.06	0.08	0.19	0.16	0.06	0.05
Female	20–29	3.38	3.24	0.00	0.06	0.19	0.62	0.00	0.21
	30–39	5.15	5.32	0.17	0.16	0.66	1.05	0.50	0.28
	40–49	3.76	5.39	0.00	0.17	0.54	0.82	0.36	0.27
	50–59	4.37	6.12	0.24	0.19	0.00	1.07	0.00	0.30
	60–69	2.59	6.14	0.29	0.20	0.29	0.61	0.00	0.21
	70–79	6.44	6.81	0.00	0.21	0.00	0.56	0.00	0.16
	Mean^b^	4.14	5.26	0.11	0.16	0.34	0.82	0.19	0.25

^a^ The simulated prevalence was from the calculation of the prevalence in each sex and age group directly from the parameter set (no simulation needed).

^b^ Mean prevalence weighted by the number of observed individuals in each age group.

### Bayesian approach to calibrating transition probabilities

A Bayesian statistical approach was adopted for this analysis [29]. The basics of such an analysis are the specification of prior distributions representing the uncertainty in the MCM parameters, and the definition of the likelihood of observing the “new data” (NHANES data in our case) conditional on the values of the model parameters. The Bayesian analysis procedure updates the prior distributions to yield likelihood-based posterior distributions of the parameters based on the new data. In the following, we describe the process of defining the priors and the likelihood contributions.

### Time-step considerations

Before the analysis, it was determined that the time step-size for the MCM shown in Fig 1 needed to be less than one year, as certain types of thyroid disturbances cause a change in thyroid disease status over a period as short as a few months [30–32]. Consequently, the annual transition probabilities discussed above were converted to quarterly probabilities, which were used in the model. Details of that conversion are presented in the supplemental materials (subheading “Conversion of annual transition probabilities to quarterly transition probabilities”).

### Prior distributions

For our prior distributions we used beta and Dirichlet distributions. Beta distributions were used for transitions from states 2 and 3, which can only transition to state 6, and Dirichlet distributions were used for transitions from states 1, 4 and 5. States 1, 4, and 5 have multiple possible transitions and a Dirichlet distributions ensures that the sum of probabilities for each state with multiple transitions is 1. The beta and Dirichlet distributions are related in that the marginal probabilities of the Dirichlet distributions are beta distributions.

The parameters for the prior distributions were assigned such that the expected values of the distributions matched the values given in Tables 1 and 2, while the variance, representing our uncertainty in the ‘true’ value, was maximized. To maximize the variance, we set the minimum parameter value of the distributions to be 1.05 which serves as a compromise between maximizing the variance and having a distribution which has a peak density that is strictly between 0 and 1. Parameter values below 1 produce distributions where the most likely value is 0 or 1, neither of which is a realistic value for a transition probability. Alternate choices of minimum parameter value of the distributions, between 1 and 1.05, while maintaining a fixed expected value, had a miniscule effect on the shape of the prior distributions.

The *a priori* annual transition probabilities for t14 and t15, which were simple multiples of incident clinical disease rates in Flynn et al. [11], reflect the expected higher risk of hypothyroidism than hyperthyroidism, and the expected higher risk of either disease in females compared with males (Table 1). The *a priori* annual transition probabilities for the remaining parameters reflect that the probability of changing states was highest for those with overt disease, and lowest for changing from subclinical states to overt states (Table 2) [12–21].

### Likelihood calculations

To update the parameters in the model, we calculated the likelihood of a set of parameters based on the comparison of the observed and simulated prevalence of thyroid disease by sex and age group. Technical details of the likelihood calculations are presented in the supplementary materials (subheading “Likelihood Calculation”). In brief, the NHANES data we used for updating the MCM parameter estimates via the Bayesian approach were prevalences by sex- and 10-year age groups. In order to compute likelihoods, we averaged the model-predicted prevalences across all the ages in a given age group, by sex. These model-predicted prevalences were obtained through analytical calculation of the prevalence at each age directly from the parameter set using matrix multiplication. For “diagnosed thyroid disease” (state 6), the contribution to the likelihood of the parameters was based on the binomial distribution. In the NHANES data, counts of thyroid disease may have included subjects with only nonfunctional thyroid disease. We estimated that 95% of cases were functional [18,33–35] and adjusted the target counts accordingly. For undiagnosed thyroid disease (states 2–5), the contribution to likelihood of parameters was based on a multinomial distribution.

### Implementation

Given the above development, both priors for MCM parameters and likelihoods associated with additional (NHANES) observations were defined. The analyses were implemented using RStan, version 2.16.2 [36], and run under the R programming language, version 3.3.1 [37].

Successful series of model iterations were obtained in all cases based on the convergence of the chains and the effective sample size for each model parameter. One iteration of the model entailed calculation of the likelihood of a given set of parameters. Three chains were used for sampling the posterior probabilities with 5,000 iterations in each chain, of which 2,500 were discarded as an initial warm-up period. This resulted in 7,500 samples for the posterior distributions. For the final prevalence values shown in the results, the maximum *a posteriori* (MAP) parameter values were used, which is the set of parameter values among the sampled values that maximized the likelihood.

### Comparison to published data on incidence rates

To compare model derived incidence rates of treated thyroid disease with published values, we generated a population using the MAP transition probability values for the MCM. To generate this population, individuals initially in the ‘normal’ state were checked for a state transition at each timestep as they aged. The probability of state transition was based on the age and sex dependent MCM transition matrix. Individuals within this population provided realistic time courses of lifetime thyroid disease status. This population consisted of 80,000 male and 80,000 female individuals, whose lifetime thyroid disease status was assigned from [0,80) years of age. The published values for comparison were those of Flynn et al. [11], who conducted a population-based dynamic cohort study of the residents of Tayside, a region of Scotland, over the period 1993–1997, during which 1.3 x 10^6^ person-years were observed. Outcome ascertainment was based on unique patient identifiers and comprehensive medical record linkage, with detailed criteria for disease classification. To compare with the results of Flynn, et al. [11], we calculated the incidence rates of treated hypothyroidism and hyperthyroidism for each age group and sex within the predicted population.

### Uncertainty and sensitivity analyses

We also examined the coefficient of variation (CV) of the MCM parameters sampled by Stan among those models with the best fit to the data (change in Bayesian Information Criteria compared to the MCM with the MAP transition probability values ≤ 4). The CV is the ratio of the standard deviation to the mean of a parameter value and communicates information about the width of the distribution of each parameter within the subset of the best samples in terms of fit. A large CV suggests a parameter that had a smaller impact on model fit, since that parameter can hold many values while the model still fits the data relatively well, while a small CV suggest a parameter is relatively limited in value for the model to fit. This evaluation was analogous to a sensitivity analysis in a deterministic model, except that in our case the values of each of the other parameters varied over all of its sampled posterior.

In additional sensitivity analyses, we repeated the Bayesian model fitting procedure after making the distributions of all *a priori* parameters uniform (or the equivalent for the Dirichlet distributed parameters), after making the distributions of all *a priori* parameters distributions have a minimum parameter value of 1.5 (decreased variance), after being more selective about studies to include in the identification of the prior distributions (alternative priors), and after changing the proportion of nonfunctional thyroid disease to 85%.

## Results

### MAP parameter values

The MAP parameter values, expressed as annual risks, for t14 and t15, continued to reflect higher risk of hypothyroidism than hyperthyroidism, and the expected higher risk of either disease in females compared with males (Table 5). Risk tended to increase less with age for the MAP as compared with the *a priori* values. The MAP parameter values at the bottom of Table 5 were generally fairly close to the *a priori* mean values. The ratios of the MAP parameter values in Table 5 to the corresponding mean *a priori* parameter values were within a 3-fold range for 29 out of the 32 parameters. One of the parameters with a more extreme ratio was for t14, for males 20–29; t14 was among the parameters with the weakest support for the *a priori* values. Another ratio with an extreme value was for t53, suggesting that a substantial proportion of subjects with subclinical hyperthyroidism develop overt disease each year. The last ratio with an extreme value was for t56, also among the *a priori* parameters with the weakest support. The MAP value of 18% for t56 suggests that a substantial portion of subjects with subclinical hyperthyroidism are treated each year. The difference between the MAP and *a priori* parameter values was greatest for t36, for which the MAP suggested about a half of patients with overt hyperthyroidism were treated each year (the prior mean was 0.16).

**Table 5:** Maximum *a posteriori* values for annual transitions.

Parameter	Description	Sex*	Age group (y)*	Mean a priori (%)	Annual MAP (%)	Ratio
t14	Normal to subclinical hypothyroidism	Male	10–19	0.03	0.02	0.7
			20–29	0.07	0.37	5.3
			30–39	0.12	0.35	2.9
			40–49	0.18	0.28	1.6
			50–59	0.34	0.46	1.4
			60–69	0.51	0.71	1.4
			70–79	0.77	0.77	1.0
		Female	10–19	0.10	0.07	0.7
			20–29	0.52	0.83	1.6
			30–39	0.97	0.72	0.7
			40–49	1.74	0.69	0.4
			50–59	2.22	1.04	0.5
			60–69	2.59	0.87	0.3
			70–79	2.53	1.43	0.6
t15	Normal to subclinical hyperthyroidism	Male	10–19	0.01	0.03	3.0
			20–29	0.02	0.03	1.5
			30–39	0.04	0.06	1.5
			40–49	0.06	0.04	0.7
			50–59	0.05	0.06	1.2
			60–69	0.08	0.09	1.1
			70–79	0.08	0.11	1.4
		Female	10–19	0.04	0.39	9.8
			20–29	0.22	0.16	0.7
			30–39	0.24	0.45	1.9
			40–49	0.26	0.27	1.0
			50–59	0.26	0.50	1.9
			60–69	0.32	0.21	0.7
			70–79	0.37	0.31	0.8
t47	Subclinical hypothyroidism to normal			8.05	7.86	1.0
t57	Subclinical hyperthyroidism to normal			5.98	3.14	0.5
t46	Subclinical hypothyroidism to treated			2.23	2.87	1.3
t56	Subclinical hyperthyroidism to treated			2.25	17.67	7.9
t42	Subclinical hypothyroidism to overt			1.88	0.79	0.4
t53	Subclinical hyperthyroidism to overt			2.80	12.05	4.3
t26	Overt hypothyroidism to treated			21.21	26.04	1.2
t36	Overt hyperthyroidism to treated			16.03	48.30	3.0

* Only normal to subclinical transitions were age and sex dependent.

### Predicted prevalences

The lifetime prevalence of diagnosed thyroid disease observed in NHANES compared to the predicted using the MAP parameters for the MCM, were similar (Table 3), with the mean observed values being slightly higher than the predicted prevalence in men (4.10% and 3.93%) and in women (15.41% and 14.48%). For undiagnosed subclinical hypothyroidism, however, the observed prevalence for men was consistently higher than predicted, with mean values of 4.18% and 2.80% (Table 4); for females the observed values tended to be lower than predicted, with mean prevalences of 4.14% and 5.26% (Table 4). For overt hypothyroidism, comparison is limited by the sparsity of the observed data, with many age groups containing one or zero cases. The predicted prevalence increased with age in both men and women. When averaged over age groups, the observed prevalences for undiagnosed overt hypothyroidism were similar, but lower, than the predicted prevalences, with values of 0.06% and 0.08% in men and 0.11% and 0.16% in women. For hyperthyroidism, both the subclinical and overt observed prevalences were affected by sparsity in the data set. For women, the observed prevalences were lower than the predicted prevalence for both subclinical and overt hyperthyroidism, with mean values of 0.34% and 0.82% for subclinical hyperthyroidism and mean values of 0.19% and 0.25% for overt hyperthyroidism. For men, the observed prevalences of hyperthyroidism between age groups were much more variable than the predicted prevalences and were slightly higher based on comparison of the average values, with 0.19% and 0.16% for subclinical hyperthyroidism, and 0.06% and 0.05% for overt hyperthyroidism.

**Fig 4:**
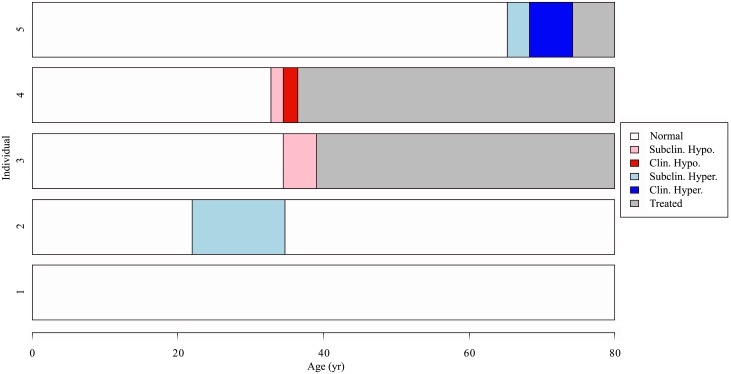
Example lifetime trajectories of thyroid disease state.

The predicted prevalences of undiagnosed thyroid disease, combined over age and sex groups, compared favorably with values from an analysis of NHANES waves not used in our analysis, with observed and predicted values of 4.1% and 3.9% for subclinical hypothyroidism; 0.1% and 0.2% for overt hypothyroidism; 0.5% and 0.2% for subclinical hyperthyroidism; and 0.2% and 0.2% for overt hyperthyroidism [27]. For this comparison, the combination of predicted prevalences over age groups was weighted by the age distribution present in Hollowell et al. [27]. Examples of functional thyroid disease status over the course of a lifetime (trajectories) are shown in Fig 4.

### Comparison to published data on incidence rates

Rates of treated hypothyroidism among males were similar between those observed by Flynn et al. [11] and our predicted values (Table 6). Rates of treated hypothyroidism among females were consistently higher in the Flynn data compared with those predicted by the model. Observed rates of treated hyperthyroidism among males and females were consistently lower than those predicted by the model.

**Table 6:** Incidence rates of treated hypothyroidism and hyperthyroidism expressed per 1000 individuals per year with 95% CI.

Sex	Age Group (y)	Hypothyroidism		Hyperthyroidism	
		Flynn et. al (11)	Predicted^a^	Flynn et. Al (11)	Predicted^a^
Male	10–19	0.09 (0.03–0.19)	0.47 (0.43–0.52)	0.03 (0.00–0.05)	0.24 (0.21–0.28)
	20–29	0.24 (0.14–0.38)	0.91 (0.85–0.98)	0.06 (0.02–0.14)	0.55 (0.50–0.60)
	30–39	0.43 (0.30–0.60)	0.94 (0.87–1.01)	0.13 (0.07–0.23)	0.48 (0.43–0.53)
	40–49	0.64 (0.48–0.84)	1.09 (1.02–1.17)	0.21 (0.13–0.32)	0.53 (0.48–0.58)
	50–59	1.19 (0.95–1.48)	1.50 (1.42–1.59)	0.18 (0.10–0.30)	0.71 (0.65–0.77)
	60–69	1.78 (1.46–2.14)	1.96 (1.87–2.07)	0.27 (0.16–0.42)	0.94 (0.87–1.01)
	70–79	2.69 (2.20–3.25)	1.97 (1.87–2.07)	0.29 (0.15–0.49)	0.91 (0.84–0.98)
Male	10–19	0.35 (0.22–0.53)	1.01 (0.95–1.09)	0.14 (0.07–0.25)	1.03 (0.96–1.10)
	20–29	1.83 (1.53–2.19)	1.95 (1.86–2.06)	0.78 (0.60–1.01)	3.40 (3.28–3.54)
	30–39	3.39 (3.01–3.82)	2.03 (1.93–2.14)	0.85 (0.68–1.06)	3.07 (2.95–3.20)
	40–49	6.07 (5.55–6.62)	2.34 (2.23–2.46)	0.90 (0.72–1.14)	3.59 (3.45–3.74)
	50–59	7.78 (7.14–8.46)	2.35 (2.24–2.47)	0.91 (0.71–1.14)	2.35 (2.24–2.47)
	60–69	9.06 (8.37–9.79)	2.56 (2.44–2.69)	1.12 (0.90–1.38)	1.91 (1.80–2.02)
	70–79	8.84 (8.08–9.65)	2.87 (2.74–3.01)	1.29 (1.03–1.60)	1.97 (1.86–2.08)

^a^ A simulated population of 80,000 men and 80,000 women was used.

### Uncertainty and sensitivity analyses

When we examined the uncertainty in the parameters, those with a large CV were sampled broadly, suggesting that the likelihood was not sensitive to changes in those parameters and that, as a result, the optimal value for those parameters was determined with less precision (Table B in S1 Supporting Information). The transitions that generally had the highest CV were the transitions from the normal to the subclinical hyperthyroidism state. Another general finding was that transitions involving hyperthyroidism had higher CV that the analogous hypothyroid transitions. This may be due to the lower prevalence of hyperthyroidism resulting in more uncertainty about those transitions.

Comparison of the MAP parameter values to the mean parameter values showed that they were generally similar, with most of the MAP values within a standard deviation of the mean (Table C in S1 Supporting Information). Plots of the posterior distributions showed that the variance in the distributions tended to be larger in women than in men (Fig. A-D in S1 Supporting Information). There did not seem to be a trend in whether the MAP was larger or smaller than the mean value.

In the additional sensitivity analysis, the MCM MAP parameter values obtained with uniform priors, compared with the values obtained with the “baseline” priors, in general varied several fold (Table D in S1 Supporting Information). For example, the value of t14 for males 70–79 years old using a uniform prior was nearly 100 times higher than the baseline value, and the value of t56 was one hundred times lower. The large effect of using uniform priors on the MAP parameter values indicated that the simulation results were influenced greatly by the use of informative priors. When we decreased the variance of the prior parameter distributions, the effect on the MAP values compared with the baseline model was much less marked, with most ratios being in the 0.5 to 1.5 range. Again, some of the parameter values were more affected than others by a change in the *a priori* distribution. Parameters t57 and t46 were more than two-fold larger with the decreased variance of the prior, and the ratio of values of t15 for females were 0.26 and 0.15 for those in the 40–49 and 50–59 year age groups.

Few studies were available to calculate the a priori estimates of transition probabilities (Table 2); these were heterogeneous in design, only one was from the U.S. [17], and none were ideal. For example, none of the studies were population-based and of incident cases at enrollment. Nonetheless, for the transitions from subclinical hypothyroidism to overt hypothyroidism (t42), and from subclinical hypothyroidism to normal thyroid state (t47), the data from two studies were possibly more informative than others because they were of cases newly referred for evaluation by an endocrinologist and thus more likely to be incident cases at baseline [12,16]. To evaluate the sensitivity of our results to the data used to estimate the a priori distribution of t42 and t47, we repeated the entire simulation with the revised inputs based on the data from Diez et al. [12] and Rosário et al. [16]. Inclusion of only these two studies resulted in prior annual transition probabilities of 0.05 for t42 and 0.06 for t47. For the other transition probabilities shown in Table 2, an obvious strength of one study vs. another was not apparent, thus no changes in input were implemented for these. This resulted in a relatively small change in the prior distributions, and the resulting MAP parameter values were generally within about a factor of 2 of the baseline simulation. When we changed the proportion of “treated” thyroid disease that was functional to 85% from 95%, the effect on the MAP values compared with the baseline model was also relatively small, with most ratios being in the 0.5–1.5 range, with just a few parameters showing more extreme ratios.

In general, the additional sensitivity analyses showed that the predicted prevalence of undiagnosed thyroid disease was not much affected by the changed inputs to the simulation (Table E in S1 Supporting Information). Changes in the variance of the *a priori* parameter distributions on average increased the predicted prevalence of treated thyroid disease for males; for females these changes in inputs slightly decreased the prevalences (Table F in S1 Supporting Information). A similar effect was seen when the alternative priors were used. As expected, decreasing the proportion of functional treated thyroid disease to 85% decreased the average prevalences of treated disease, but did not have a strong effect on undiagnosed thyroid disease. An exception to this was in subclinical hypothyroidism among females, in which the prevalence was lower in most age groups and the average prevalence across age was slightly better matched to the observed values. Overall, the additional simulations showed that changes in the simulation conditions had larger effects on the MAP parameter values than on the predicted prevalences.

## Discussion

Our model predicted the prevalence of diagnosed thyroid disease for a given sex and age fairly well. The predicted prevalence of undiagnosed subclinical hypothyroidism, however, was higher than observed for women and lower than observed for men. The predicted values for the other categories of undiagnosed disease were fairly close to observed and better fits might have been compromised by the rarity of the outcome, causing many zeros in the target tables. Our mean predicted prevalences of undiagnosed functional disease were similar to reported values in another NHANES-based study, based on data from 1988–1994 [27], and to values from a survey in Colorado, which used slightly different definitions of disease [38]. The MAP transition probabilities from one state to another were also reasonably close to *a priori* values in those instances where the *a priori* values were well supported by data.

Our model predicted incidence rates of treated thyroid disease that were of the same magnitude as published values [11,19], with 82% of the predicted values within a 4-fold range of the published values. The differences seemed to be systematic, with comparable incidence rates in hypothyroid males, underestimated incidence rates in hypothyroid females, and overestimated incidence rates in hyperthyroid males and females. This may be due to differences in the sources of the data; while the data used to fit the model was from a population in the U.S., the data used for incidence rates was based on a population from Scotland. WHO has classified the U.S. as being “at risk of iodine-induced hyperthyroidism”, and recently classified the U.K. as mildly iodine deficient [39,40]. Iodine deficiency is a risk factor for hypothyroidism [41]. Thus, our results are broadly consistent with what is known about national iodine status and its relation to disease etiology.

When the model fitting procedure identified the parameter values giving the maximum likelihoods, much weight was given to the parameter values that would give a good fit to the number of subjects in state 6 because the numbers of individuals in state 6 was greater than the numbers in the other non-normal states. Thus, it was "more important" for the model to fit the state 6 observations than to fit the other observations. More women than men were in the non- normal states. For the next most frequent state (4, subclinical hypothyroidism), the model fit was not as good, and the fit for women was better than for men, reflecting the greater influence of women in determining parameter estimates.

The annual transition rates predicted by the MCM for going from overt thyroid disease to treated (hypothyroidism, 22%, hyperthyroidism, 48%) seemed surprisingly low. However, patients seen clinically are probably more symptomatic than cases identified by screening the general population, such as in NHANES. A large screening study of the general population, with follow-up to ascertain treatment status, would be a useful way to assess the validity of the predictions.

If the results of the model evaluation were used to identify data that would most likely improve the MCM in terms of its ability to replicate disease prevalence, better *a priori* probability estimates for development, treatment, and reversion of hypothyroid disease would be the most valuable. On the other hand, if the goal were to improve the precision of the specific transition rates in the model, additional information on the development of subclinical hyperthyroidism would be of the most benefit. Another piece of useful data would be information within the treated population on whether they were diagnosed with a hypo- or hyper-thyroid condition. This would allow for splitting of the ‘treated’ state into two states in the model and better restrict the model parameterization by limiting the ways that the prevalence of the ‘treated’ state can be met.

A complete evaluation of our model would involve comparing how the predicted distribution of lifetime trajectories compares with a distribution of observed trajectories. Data to validate this aspect of our model could be obtained if a large population had annual clinical screening for thyroid disease, and measurement of thyroid hormones or storage of blood specimens for future studies.

Finer stratification of age groups might have resulted in improved model fits, especially for women in their twenties. Note that all the transition probabilities in the model probably are age- and sex- dependent, and that our simplifying assumption of homogeneity for transitions other than t14 and t15 in our model might have resulted in inaccuracies. However, estimation of additional age-specific parameters might have resulted in a model with too many parameters for the Bayesian procedure to converge.

In the studies of the natural history of thyroid disease summarized in Table 2, the eligibility criteria and definitions of disease state varied somewhat. This meant the annual transition probabilities calculated from each study were for slightly different conditions. Furthermore, sex and age distributions across the study populations varied, and overall, they differed from NHANES, meaning that the transition probability summaries may not have been optimally weighted. In addition, the subjects in such studies might not be typical in that they were from case series at referral centers, and their thyroid disease state might have had unrepresentatively long duration in order to have been enrolled. These uncertainties supported the choice of relatively flat prior distributions.

Different subtypes of thyroid disease each have their own pathophysiology, descriptive epidemiology, and natural history. Subtype specific models would be ideal for applying the simulated results to the clinical setting. In our model, however, the goal was to model progression from one phenotypic disease state to another, based on TSH concentrations and questionnaire responses, independent of pathophysiology. The thyroid hormone data and “ever thyroid disease” question available to us from NHANES, used in our target tables, allowed only phenotypic categorization, and few of the data used for a priori parameter estimates had information that was specific to a given pathophysiology.

The disease state classification in our target tables and in our model differed somewhat. The recommended criteria for clinical diagnosis of thyroid disease is based on hormone concentrations and symptoms [24,42]—though variation in diagnostic criteria appears to be frequent (Table 2). This means that in NHANES, for example, a patient with a high TSH and a normal fT4 with no thyroid symptoms might nonetheless have been told by their physician that they had subclinical hypothyroidism, and therefore would have reported having been diagnosed, which would put them in state 6 in a target table. Our model, however, was based on biochemical criteria only (after excluding those with a diagnosis of thyroid disease), and we assumed that everyone with biochemical hypothyroidism was in state 4. In the model we assumed the subjects in states 2–5 were not sufficiently symptomatic as to require treatment or had not yet been seen by a physician. If a large proportion of those with subclinical disease have never been diagnosed as such, which may be the case [27,38], the misclassification in the target table may have been minimal. A related issue was that the best short label for state 6 (“treated”) did not capture the nuances of that state definition. In the observed data, categorization was based on a reported diagnosis of thyroid disease. An alternative label might have been “diagnosed”, except that categorization into states 2–5 in the a priori data was based on diagnosis by a physician of untreated, asymptomatic disease. We used “treated” as a label for state 6 so that the model would reflect the spectrum and progression of disease severity. Improved population data on thyroid disease history could help make classification of state agree better between the target tables and the model but may be difficult to obtain.

Furthermore, when NHANES subjects reported ever having had thyroid disease, they would have included nonfunctional thyroid disease such as euthyroid goiter and thyroid nodules, conditions not predicted in the model. Among those reporting thyroid disease, if the proportion of nonfunctional disease was different than 5%, the model results might have been affected. Use of a greater number of thyroid disease states in the model could address these issues, but we would not have been able to construct corresponding target tables, and priors would have been difficult to support with data. NHANES data on symptoms that might be due to thyroid disease, and on the history of nonfunctional disease, were not sufficient to allow more detailed characterization of thyroid state. Thus, perfect correspondence between the disease states in the target tables and the model was not attainable.

Our model assumes that any given transition was independent of prior thyroid disease history. Some patients with thyroiditis may swing from hyperthyroidism to hypothyroidism, and no corresponding pathway existed for such patients in our simple model. The occurrence of such trajectories, especially among patients who are not treated, however, may be relatively rare [43]. If, when predicting transitions, our model took into account the history of previous disease states rather than just the current state, it might have better reflected reality and potentially improved the model fit. Alternative modeling approaches that can do this are higher order Markov chain, variable order Markov chain, and recurrent neural networks. Any of the alternatives would involve a more complex model with additional parameters; the impact of those elaborations in terms of model fit would have to be carefully evaluated, with no guarantee of improved model performance. Moreover, unlike most of the parameters in our model, for the additional or revised parameters no data are available to assign a priori probability distributions. Furthermore, the alternative approaches would be less parsimonious and cost-effective to develop. As more detailed data on the natural history of thyroid disease becomes available, these alternative approaches would be worth considering.

Inclusion of additional transition probabilities in the model, such as t74 and t35, could potentially make it more realistic and improve the model fit. However, no data were available to inform a priori parameter distributions, and such transitions may be rare. Another potential future refinement of the model would be to add a death state, as hyperthyroid states slightly increase the relative risk of death [44] which could affect prevalence.

The MCM that we have developed has several potential advantages and uses. First, it will support simulations of epidemiologic data on the relation of thyroid disease to serum concentrations of environmental contaminants [45,46]. The simulations will allow a quantitative bias analysis of these cross-sectional studies [47]. These epidemiologic studies focused on environmental contaminants as etiologic agents rather than on how thyroid disease might lead to less renal excretion and higher serum concentrations. The simulations will enable an assessment of whether the reported association can be explained on the basis of pharmacokinetics and reverse causality. The differences between the observed and model-predicted prevalence of subclinical hypothyroidism noted above may not have an important effect on the results of the quantitative bias analysis. In addition, the estimates of the incidence of treated thyroid disease in the U.S. may themselves be useful. Relatively few reports exist on the incidence of thyroid disease in the U.S., and the ones that are available tend to have limitations such as being applicable to one sex or age group, specificity to a disease subtype such as Graves’ disease, small sample size, or uncertain applicability to the present time [48–51]. Estimates of the incidence of treated disease in the U.S. might be important for health care resource allocation. Estimates of the probability of a patient’s disease regressing or progressing may be useful for patient management, including informing patients of the approximate frequency of each possibility.

## Supporting information

S1 Supporting Information Additional technical details and results from sensitivity analysis. (DOCX)

S1 Supporting Data R and Stan code for the MCM and Markov chain results. (ZIP)
