# Supplementary material for: A model of functional thyroid disease status over the lifetime
Source: PLoS One. 2019 Jul 18;14(7):e0219769. doi: 10.1371/journal.pone.0219769 (PMC6638952; doi:10.1371/journal.pone.0219769)
Supplement: S1 Supporting Information — (DOCX) [file pone.0219769.s001.docx]

A model of functional thyroid disease status over the lifetime

Supporting Information

Michael W. Dzierlenga^1,#a*^, Bruce C. Allen^2^, Peyton L. Ward^3^, Harvey J. Clewell III^3^, Matthew P. Longnecker^3^

^1^ScitoVation, Research Triangle Park, North Carolina, United States of America

^2^Independent Consultant, Chapel Hill, North Carolina, United States of America

^3^Ramboll, Research Triangle Park, North Carolina, United States of America

^#a^Current address: Ramboll, Research Triangle Park, North Carolina, United States of America

*Corresponding author

E-mail: mdzierlenga@ramboll.com

**Supporting Information**

**Conversion of annual transition probabilities to quarterly transition probabilities**

Consider the matrix of Fig 2 to be the matrix of quarterly rates of interest to us. First note that t66 and t77 both equal 1: States 6 and 7 are terminal states such that when those states are achieved, there is no transition away from them.

Next consider the rows 2 and 3: once in an Overt state the only transition to a different state will be to State 6, Treated. If A is the matrix of annual transition rates (with entries aij), then it is easy to show that

$$t22 = \sqrt[4]{a22}$$

$$t33 = \sqrt[4]{a33}$$

$tk6 = 1- tkk (k = 2,3)$.

I.e., the desired quarterly rates are completely determined by the annual rates a22 and a33. Once we define priors for a22 and a33, which we can do from the cited prior data on annual transitions (see Table 2), then the priors for t22, t33, and therefore for t26 and t36, are determined and can be used in the Bayesian updating. Beta priors for a26 and a36 (from which we get a22 and a33 by subtraction) were used, with parameter values as shown in Table 2 (main paper).

For row 1 of the matrix, the issue is somewhat more complicated, because of the fact that there are non-zero quarterly transitions from State 1 to both State 4 and State 5 (Subclinical states). Note, first of all, that this entails that the annual transition rates from State 1 to any other state are non-zero (over the course of four quarterly transitions, State 1 can “end up” in any other state). Note, however, that we can consider a matrix of the form

$$\begin{matrix} x & y & z \\ 0 & 1 & 0 \\ 0 & 0 & 1 \end{matrix}$$

to represent annual transitions from State 1 to either “hypothyroidism” (y) or “hyperthyroidism” (z). By “hypothyroidism” we mean Subclinical Hypothyroidism, Overt Hypothyroidism, returned-to-Normal (State 7) or Treated. Similarly for “hyperthyroidism.” Once transitioned from Normal, the state will never return to Normal, and it will stay “hypothyroidism” or “hyperthyroidism.” As always in a transition matrix, $x + y+ z = 1.$ As shown in the supplemental material, if we interpret the annual data available (1) to represent annual rates of transition away from Normal (regardless of what state it ends at), then we can again solve for the values of the quarterly transition matrix of interest, specifically t11, t14, and t15, as follows:

$$t11 = \sqrt[4]{x}$$

$$t14 = (1-\sqrt[4]{x})*\frac{y}{(y+z)}$$

$$t15 = (1-\sqrt[4]{x})*\frac{z}{(y+z)}$$

It should be noted here that one cannot simply treat y and z with independent Beta prior distributions. They are not independent and in fact (with x) must sum to one. Therefore, the age- and sex-specific priors used were defined as Dirichlet distributions, each such distribution defining the joint distribution of x, y and z on [0,1]^3^. The marginal distribution for x, given that x, y, and z are Dirichlet distributed, is a Beta distribution (and similarly for y and z). The parameters (α(i), i= 1, 2, 3) of that Dirichlet were selected so that the marginal expected values were equal to the best estimates based on the Flynn et al. [1] data, with the added stipulation that the minimums of the two parameters of the marginal Betas were greater than or equal to 1.05, as discussed above for Beta distributions.

Finally, for rows 4 and 5, we similarly want Dirichlet distributions as priors for the transitions on each of those rows. The issue becomes more complicated because there are four transition on each of those rows and the relationship between the annual transitions for which we have data, and the quarterly transitions of interest is not straight-forward [2]. Rather, we defined priors directly for the quarterly rates, following this procedure.

Given a set of “best estimates” of annual transition rates (say a42, a44, a46, a47; the case for State 5 transitions is the same), we again use a Dirichlet distribution that has the associated marginal means equal to

$$t44: \sqrt[4]{a44}$$

$$t42: (1-\sqrt[4]{a44})*\frac{a42}{(a42 + a46+a47)}$$

$$t46: (1-\sqrt[4]{a44})*\frac{a46}{(a42 + a46+a47)}$$

$$t47: (1-\sqrt[4]{a44})*\frac{a47}{(a42 + a46+a47)}$$

Given those means, we again picked the Dirichlet prior parameters such that the minimum of the marginal parameters was 1.05, “maximizing” the prior variability under the constraint of curve shapes that are not U- or J-shaped.

**Likelihood Calculation**

Given the above, and for any particular sample of MCM parameters, we have a total of 14 distinct quarterly transition matrices, two sets of 7 for each sex and age range (10-19 through 70-79). We enumerate those matrices as M[s,r], with s (= 1,2) representing sex and r (= 1, …, 7) representing the age range. Multiplying an initial state vector, v^1^, (which we assume to be 100% normal individuals at age 10) by the product of the matrices up to the age of interest defines the prevalence of each of the seven states at that age. For example, the vector of prevalences among males (s=1) at age 11 (in age range 1) would be

$$\mathrm{prev}[1,11] = v* {M[1,1]}^{4}$$

and for age 24 would be

$$\mathrm{prev}[1,24] = v* {M[1,1]}^{40}* {M[1,2]}^{16}.$$

Note the powers on the matrices are in multiples of 4 (for integer-valued ages), because the M[i,j] matrices are quarterly transition matrices (four matrix multiplications per year).

The NHANES data we used for updating via the Bayesian approach present prevalences by sex and 10-year age ranges. In order to compute likelihoods, we averaged the model-predicted prevalences across all the ages corresponding to the age range in question. So, for example, predicted female prevalences for the age range 20-29 were averaged over the 40 products that constitute the predicted prevalences for females in that age range. In essence, this procedure assumes that the observed prevalences were obtained, in this example, from females who were equally distributed across quarter-year ages from 20 to 29.

Given those model-predicted prevalences, we could define the likelihood given the observations under consideration. The NHANES data were reported in one of two ways, as described above. First, there were counts of thyroid disease cases among all (sex-specific) observations within an age range. We interpreted “thyroid disease cases” as those being in State 6 (Treated) in the MCM. The likelihood given such observations was based on the binomial distribution, with probability of observing State 6 being the (averaged) prevalence rate for the sex and age-range under consideration. In the NHANES data, counts of thyroid disease may have included subjects with nonfunctional thyroid disease. We estimated that 95% of cases were functional [3–6] and adjusted the target counts accordingly.

The second type of observation was a count of individuals in States 1 - 5 or 7 (i.e., not Treated) among those known not to be Treated. Such counts arise from a multinomial distribution. However, because those individuals were known not to be Treated, one must compute the conditional probabilities (prevalences) given that fact. Extending the notation from above, that probability is equal to

$$\mathrm{prev}'[s,r,j] = \mathrm{prev}[s,r,j]/(1 - \mathrm{prev}[s,r,6])$$

where $\mathrm{prev}[s,r,j]$ is the model-predicted prevalence (averaged within sex s and age-range r) of state *j*. For these likelihood contributions, the observations of “Normal” were considered to be those that were in State 1 or State 7 (i.e., $\mathrm{prev}'[s,r,1] + \mathrm{prev}'[s,r,7]$ was the conditional probability associated with the observations that were designated to be in a normal state).

**Supporting Tables**

**Table A. Thyroid disease drugs used for classification of diagnosed thyroid disease.**

| NHANES Drug Code | NHANES Name |
| --- | --- |
| a55820 | Thyroglobulin |
| c00103 | Thyroid Hormones - Unspecified |
| d00278 | Levothyroxine |
| d00290 | Methimazole |
| d00361 | Propylthiouracil |
| d00655 | Thyroid Desiccated |
| d00658 | Liothyronine |
| d05717 | Sunitinib |
| h00019 | Levothyroxine; Liothyronine |

The drugs in the table were those with a Multnum Lexicon Therapeutic Classification Scheme second level category name of “thyroid hormone” or “antithyroid agent”; in addition, thyroglobulin was included because it is sometimes used to treat hypothyroidism.

**Table B. Coefficient of variations for the *a posteriori* transition probability distributions (annual transitions) for set of samples with likelihood close to the MAP value (≤ 4 BIC).**

| Parameter | Description | Sex^*^ | Age  group (y)^*^ | CV (%) |
| --- | --- | --- | --- | --- |
|  |  |  |  |  |
| t14 | Normal to subclinical hypothyroidism | Male | 20-29 | 13.4 |
|  |  |  | 30-39 | 23.4 |
|  |  |  | 40-49 | 25.6 |
|  |  |  | 50-59 | 20.2 |
|  |  |  | 60-69 | 18.4 |
|  |  |  | 70-79 | 39.3 |
|  |  | Female | 20-29 | 16.6 |
|  |  |  | 30-39 | 24.6 |
|  |  |  | 40-49 | 21.1 |
|  |  |  | 50-59 | 19.6 |
|  |  |  | 60-69 | 20.5 |
|  |  |  | 70-79 | 56.4 |
|  |  |  |  |  |
| t15 | Normal to subclinical hyperthyroidism | Male | 20-29 | 43.8 |
|  |  |  | 30-39 | 31.3 |
|  |  |  | 40-49 | 62.7 |
|  |  |  | 50-59 | 65.0 |
|  |  |  | 60-69 | 40.4 |
|  |  |  | 70-79 | 17.2 |
|  |  | Female | 20-29 | 50.6 |
|  |  |  | 30-39 | 24.9 |
|  |  |  | 40-49 | 41.3 |
|  |  |  | 50-59 | 56.4 |
|  |  |  | 60-69 | 56.7 |
|  |  |  | 70-79 | 55.5 |
|  |  |  |  |  |
| t47 | Subclinical hypothyroidism to normal |  |  | 12.1 |
| t57 | Subclinical hyperthyroidism to normal |  |  | 38.9 |
| t46 | Subclinical hypothyroidism to treated |  |  | 17.4 |
| t56 | Subclinical hyperthyroidism to treated |  |  | 27.4 |
| t42 | Subclinical hypothyroidism to overt |  |  | 33.1 |
| t53 | Subclinical hyperthyroidism to overt |  |  | 33.8 |
| t26 | Overt hypothyroidism to treated |  |  | 29.0 |
| t36 | Overt hyperthyroidism to treated |  |  | 24.4 |

**Table C. Mean annual transition rates with comparison to the annual MAP values.**

| Parameter | Description | Sex^*^ | Age  group (y)^*^ | Mean ± sd (%) | MAP (%) |
| --- | --- | --- | --- | --- | --- |
|  |  |  |  |  |  |
| t14 | Normal to subclinical hypothyroidism | Male | 10-19 | 0.046 ± 0.024 | 0.016 |
|  |  |  | 20-29 | 0.319 ± 0.066 | 0.371 |
|  |  |  | 30-39 | 0.296 ± 0.097 | 0.345 |
|  |  |  | 40-49 | 0.315 ± 0.117 | 0.277 |
|  |  |  | 50-59 | 0.514 ± 0.136 | 0.462 |
|  |  |  | 60-69 | 0.637 ± 0.176 | 0.707 |
|  |  |  | 70-79 | 0.823 ± 0.226 | 0.774 |
|  |  | Female | 10-19 | 0.187 ± 0.118 | 0.070 |
|  |  |  | 20-29 | 0.829 ± 0.209 | 0.830 |
|  |  |  | 30-39 | 0.693 ± 0.240 | 0.720 |
|  |  |  | 40-49 | 0.794 ± 0.247 | 0.694 |
|  |  |  | 50-59 | 1.059 ± 0.318 | 1.041 |
|  |  |  | 60-69 | 1.023 ± 0.338 | 0.866 |
|  |  |  | 70-79 | 1.888 ± 0.587 | 1.430 |
|  |  |  |  |  |  |
| t15 | Normal to subclinical hyperthyroidism | Male | 10-19 | 0.016 ± 0.011 | 0.029 |
|  |  |  | 20-29 | 0.027 ± 0.018 | 0.033 |
|  |  |  | 30-39 | 0.042 ± 0.024 | 0.062 |
|  |  |  | 40-49 | 0.025 ± 0.021 | 0.043 |
|  |  |  | 50-59 | 0.029 ± 0.026 | 0.061 |
|  |  |  | 60-69 | 0.061 ± 0.042 | 0.089 |
|  |  |  | 70-79 | 0.045 ± 0.043 | 0.112 |
|  |  | Female | 10-19 | 0.303 ± 0.077 | 0.386 |
|  |  |  | 20-29 | 0.116 ± 0.095 | 0.156 |
|  |  |  | 30-39 | 0.460 ± 0.150 | 0.453 |
|  |  |  | 40-49 | 0.278 ± 0.171 | 0.273 |
|  |  |  | 50-59 | 0.203 ± 0.165 | 0.503 |
|  |  |  | 60-69 | 0.202 ± 0.156 | 0.205 |
|  |  |  | 70-79 | 0.166 ± 0.156 | 0.306 |
|  |  |  |  |  |  |
| t47 | Subclinical hypothyroidism to normal |  |  | 6.938 ± 1.442 | 7.860 |
| t57 | Subclinical hyperthyroidism to normal |  |  | 4.608 ± 2.484 | 3.140 |
| t46 | Subclinical hypothyroidism to treated |  |  | 4.193 ± 0.915 | 2.871 |
| t56 | Subclinical hyperthyroidism to treated |  |  | 15.265 ± 5.421 | 17.670 |
| t42 | Subclinical hypothyroidism to overt |  |  | 0.906 ± 0.540 | 0.785 |
| t53 | Subclinical hyperthyroidism to overt |  |  | 9.473 ± 4.389 | 12.052 |
| t26 | Overt hypothyroidism to treated |  |  | 36.402 ± 17.566 | 26.045 |
| t36 | Overt hyperthyroidism to treated |  |  | 35.892 ± 14.227 | 48.305 |

**Fig. A. Posterior distributions for annual normal to subclinical hypothyroidism transition probabilities.** Map values are shown as solid black lines, and mean values are shown as red dashed lines.

**
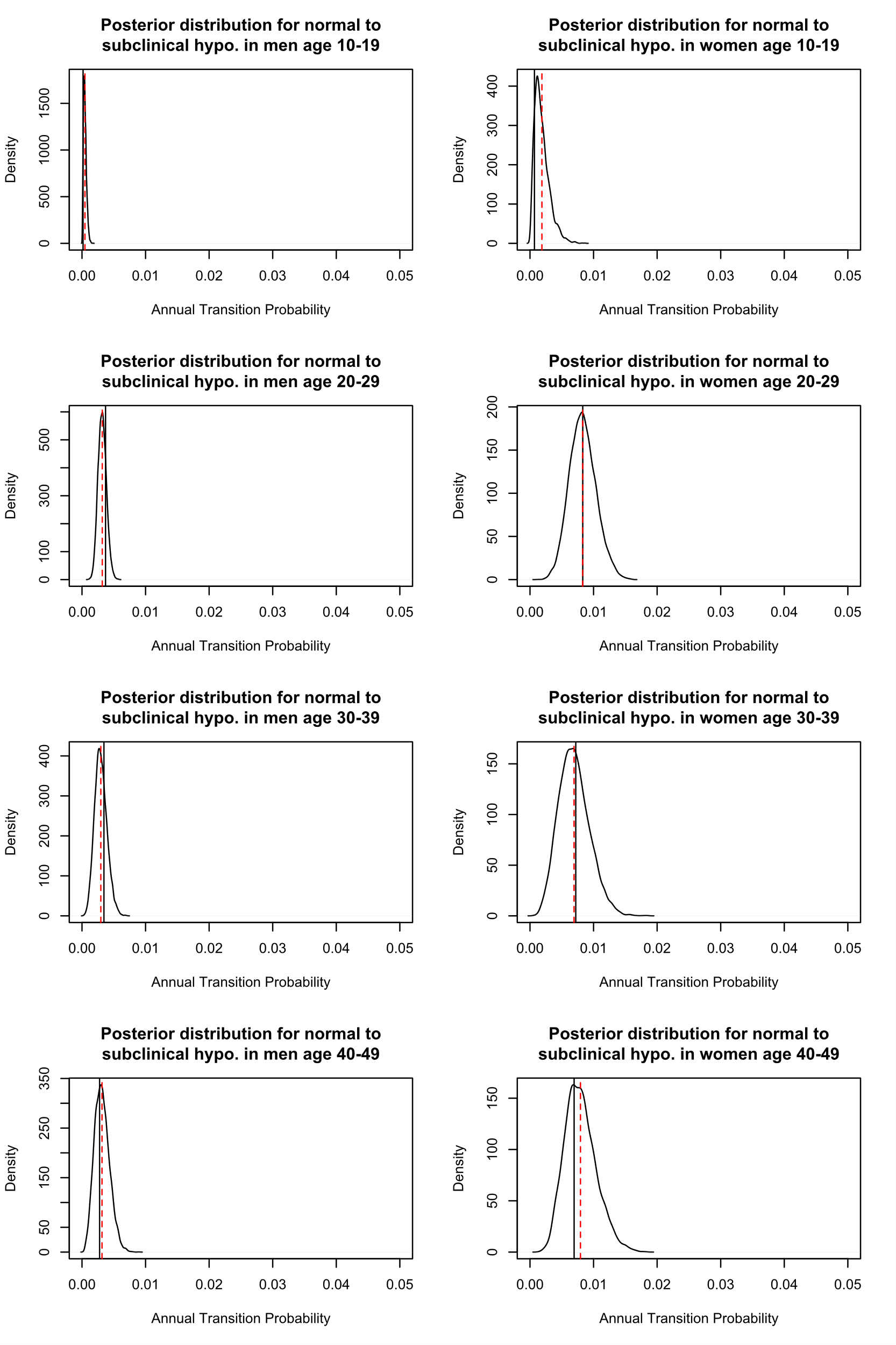
**

**
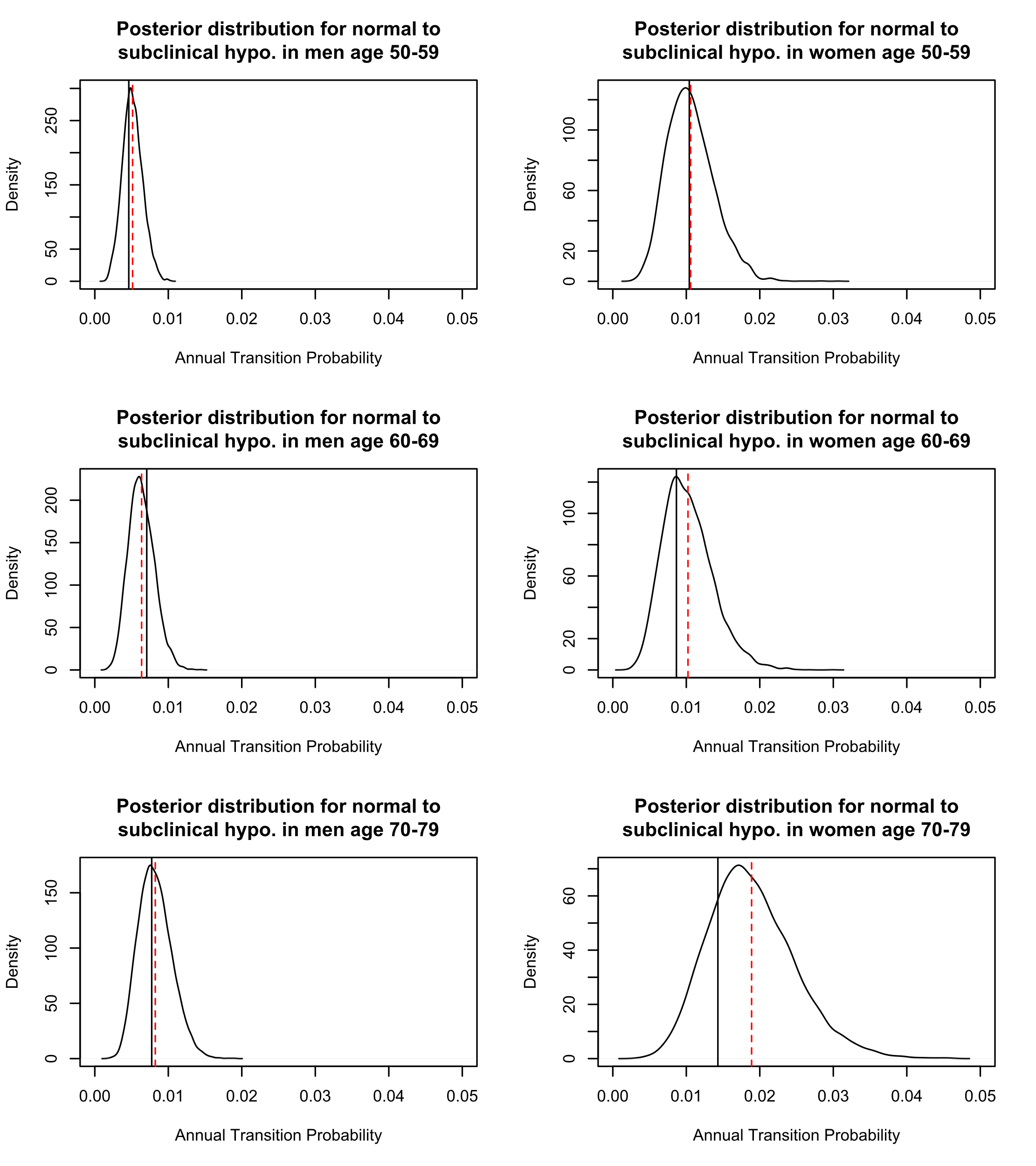
**

**
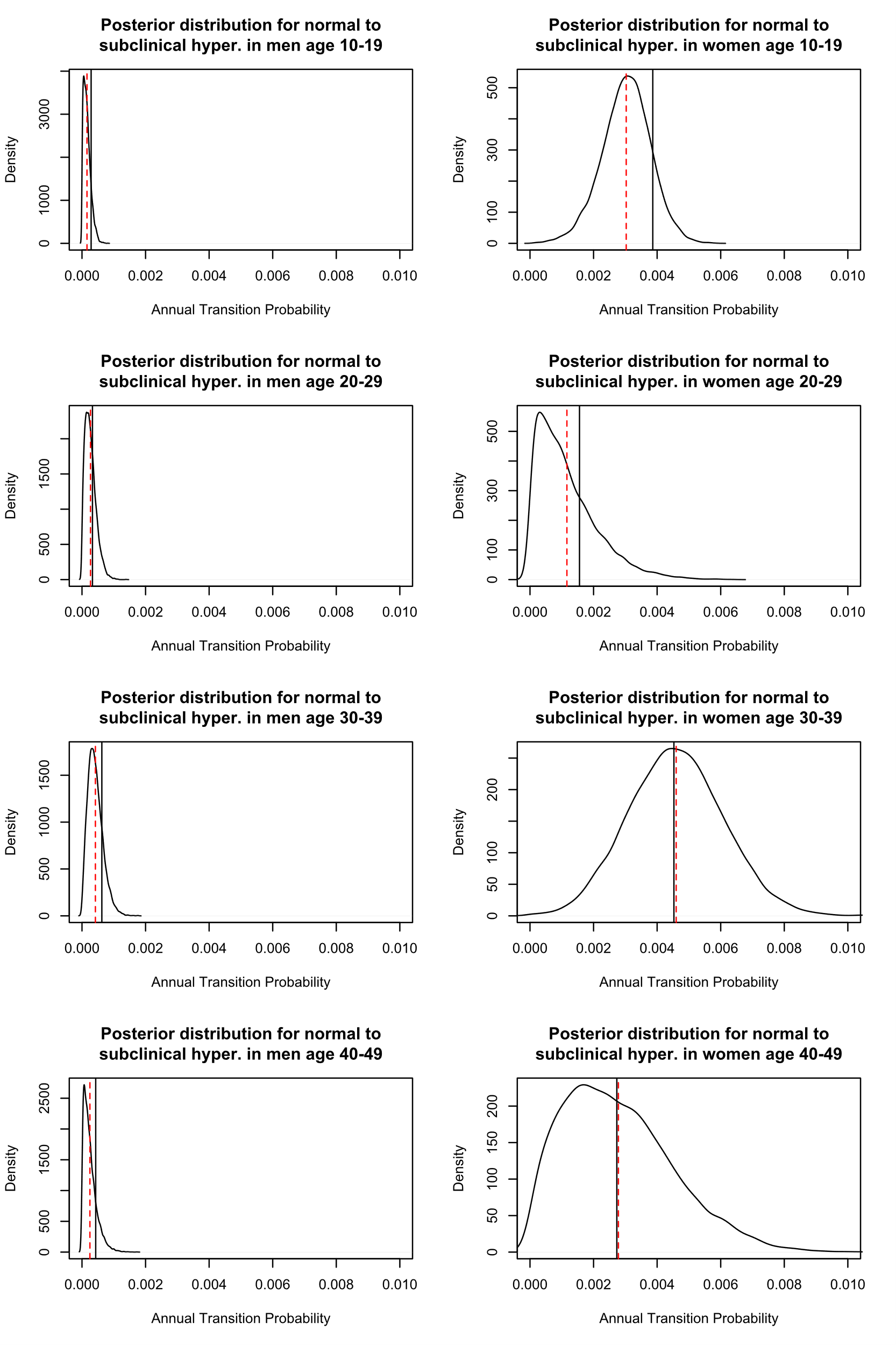
Fig. B. Posterior distributions for annual normal to subclinical hyperthyroidism transition probabilities.** Map values are shown as solid black lines, and mean values are shown as red dashed lines.

**
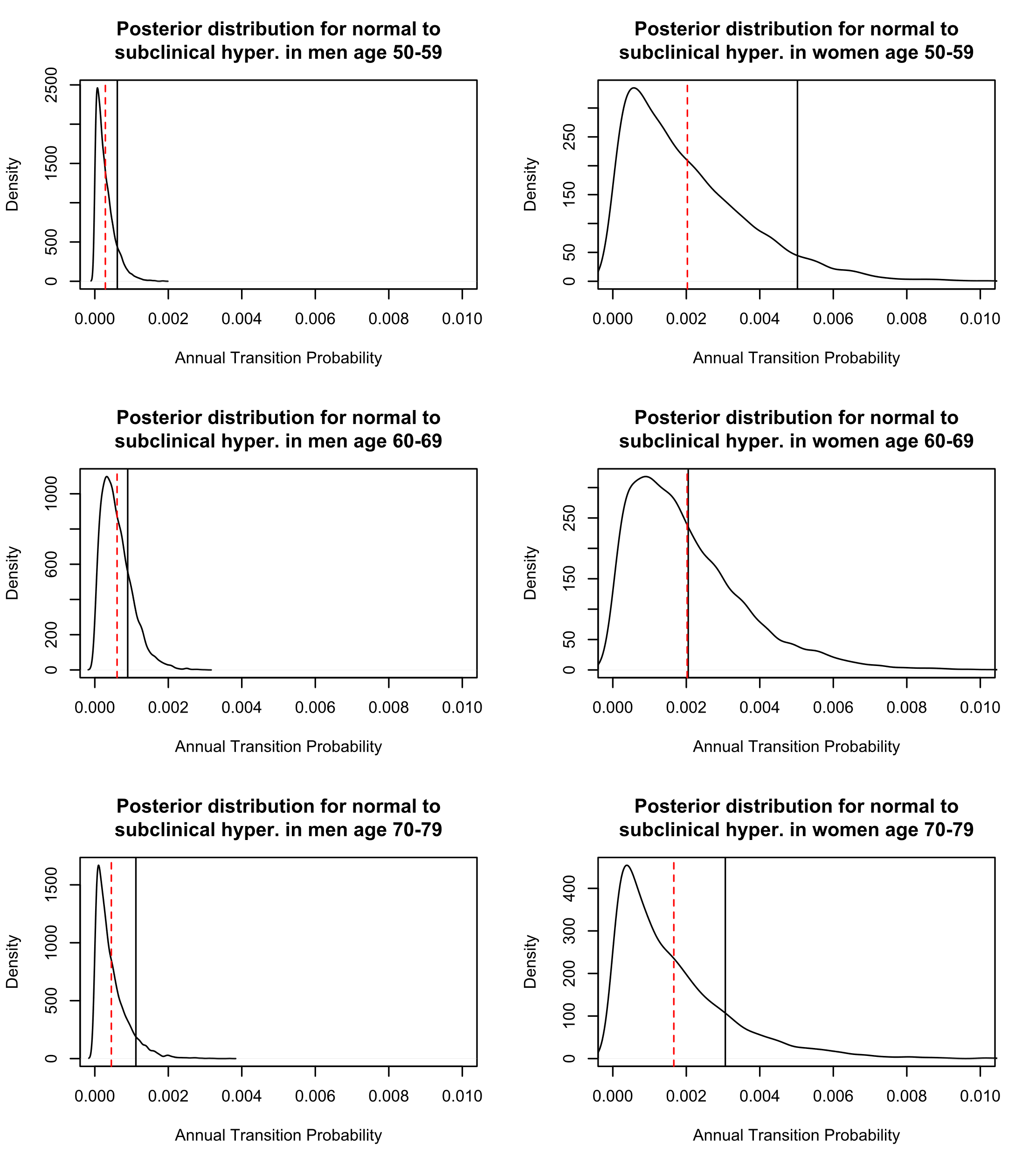
**

**Fig. C. Posterior distributions for annual transition probabilities from subclinical disease to other states.** Map values are shown as solid black lines, and mean values are shown as red dashed lines.

**
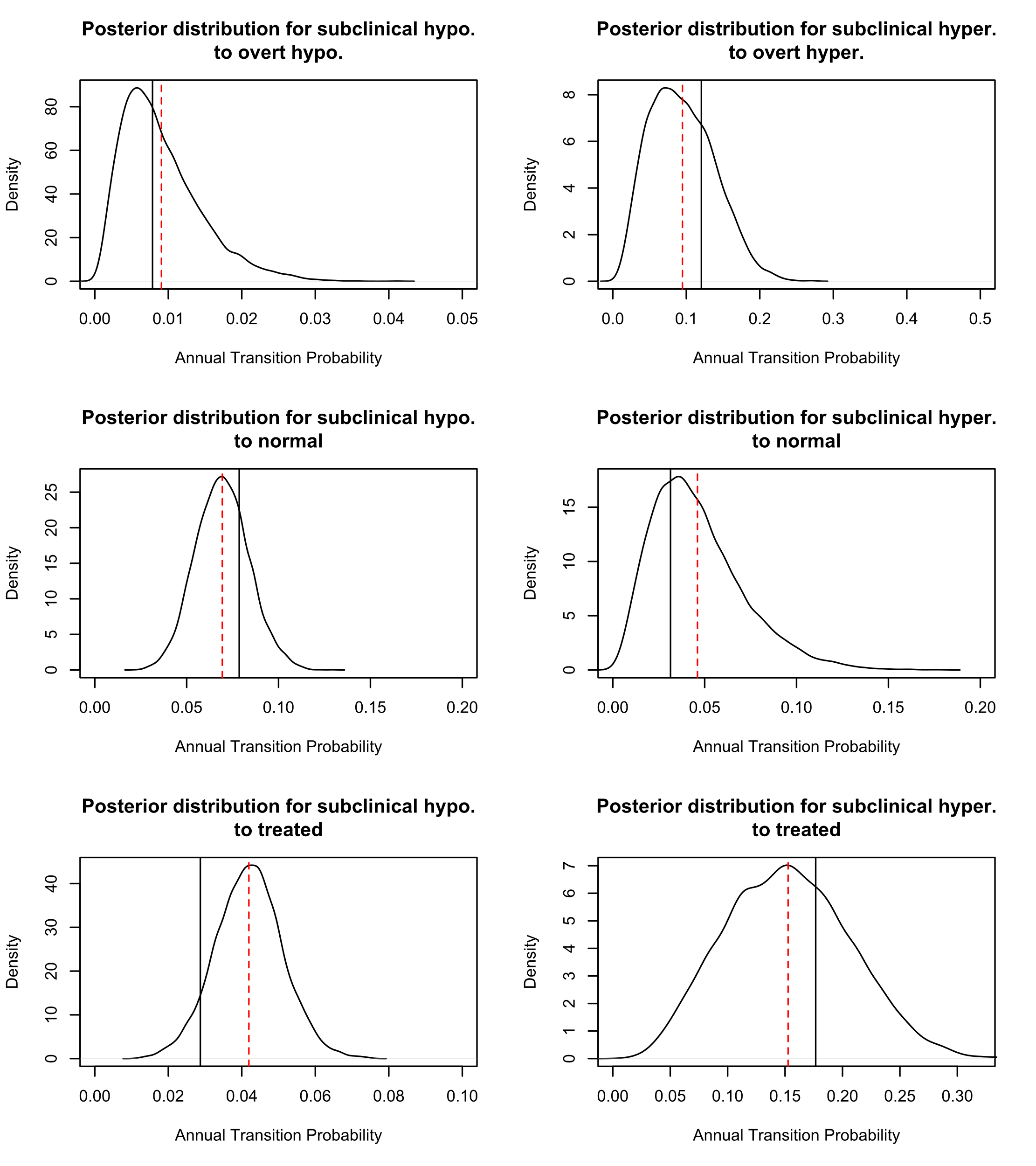
**

**Fig. D. Posterior distributions for annual transition probabilities from overt thyroid disease to the treated state.** Map values are shown as solid black lines, and mean values are shown as red dashed lines.

**
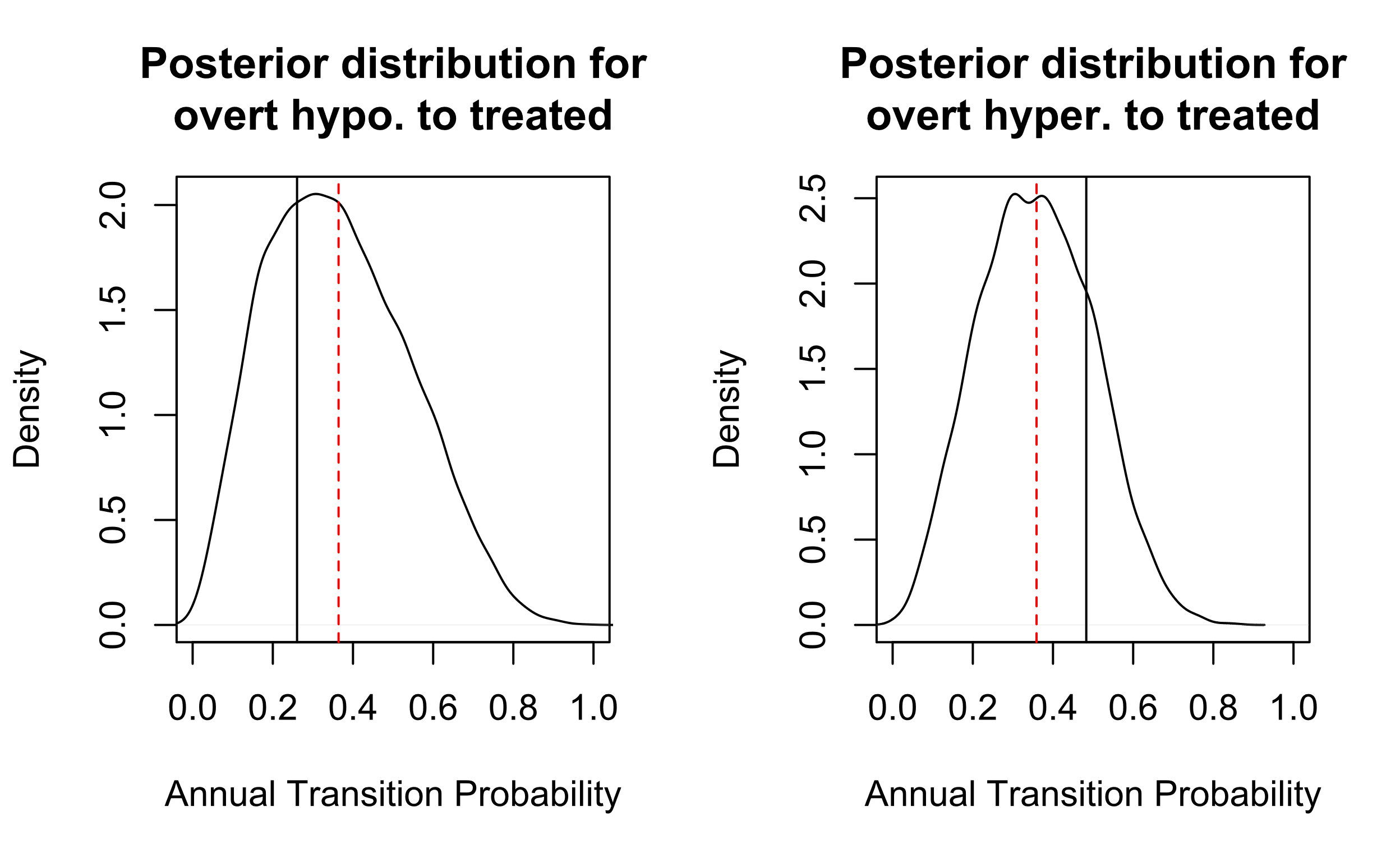
**

**Table D. Relative a posteriori values MAPs following sampling with different prior distributions or assumptions of functional disease (given as ratios between baseline and alternative annual chance of transition).**

| Parameter | Description | Sex^*^ | Age  group (y)^*^ | Uninformative Priors | Low Var. Priors | Alternative Priors | Assumption of 85% Functional Disease |
| --- | --- | --- | --- | --- | --- | --- | --- |
| t14 | Normal to subclinical hypothyroidism | Male | 20-29 | 2.79 | 0.68 | 1.18 | 1.02 |
|  |  |  | 30-39 | 1.88 | 0.74 | 1.22 | 0.98 |
|  |  |  | 40-49 | 2.42 | 1.28 | 0.57 | 0.96 |
|  |  |  | 50-59 | 10.95 | 0.78 | 0.82 | 1.30 |
|  |  |  | 60-69 | 43.57 | 0.94 | 1.30 | 0.88 |
|  |  |  | 70-79 | 94.15 | 1.20 | 0.83 | 1.20 |
|  |  | Female | 20-29 | 0.62 | 1.00 | 0.90 | 0.68 |
|  |  |  | 30-39 | 3.07 | 1.22 | 1.59 | 1.30 |
|  |  |  | 40-49 | 2.26 | 1.31 | 0.85 | 1.09 |
|  |  |  | 50-59 | 11.53 | 1.44 | 0.94 | 1.02 |
|  |  |  | 60-69 | 16.48 | 1.65 | 0.75 | 0.59 |
|  |  |  | 70-79 | 11.56 | 1.18 | 1.12 | 1.28 |
| t15 | Normal to subclinical hyperthyroidism | Male | 20-29 | 18.72 | 0.64 | 1.07 | 0.79 |
|  |  |  | 30-39 | 18.88 | 0.35 | 0.60 | 0.75 |
|  |  |  | 40-49 | 27.35 | 0.89 | 2.02 | 0.52 |
|  |  |  | 50-59 | 2.55 | 0.34 | 2.66 | 0.05 |
|  |  |  | 60-69 | 37.35 | 0.63 | 0.80 | 0.92 |
|  |  |  | 70-79 | 8.39 | 0.70 | 2.08 | 0.29 |
|  |  | Female | 20-29 | 3.36 | 0.71 | 1.28 | 0.84 |
|  |  |  | 30-39 | 2.66 | 1.04 | 0.79 | 0.81 |
|  |  |  | 40-49 | 4.98 | 0.26 | 0.62 | 1.83 |
|  |  |  | 50-59 | 2.69 | 0.15 | 1.08 | 0.38 |
|  |  |  | 60-69 | 25.73 | 1.24 | 1.33 | 1.61 |
|  |  |  | 70-79 | 14.08 | 1.17 | 6.31 | 0.72 |
| t47 | Subclinical hypothyroidism to normal |  |  | 0.13 | 0.91 | 1.05 | 1.07 |
| t57 | Subclinical hyperthyroidism to normal |  |  | 31.66 | 2.93 | 0.51 | 1.85 |
| t46 | Subclinical hypothyroidism to treated |  |  | 1.79 | 2.14 | 1.21 | 0.99 |
| t56 | Subclinical hyperthyroidism to treated |  |  | 0.01 | 0.53 | 0.98 | 0.94 |
| t42 | Subclinical hypothyroidism to overt |  |  | 1.94 | 0.87 | 0.48 | 1.55 |
| t53 | Subclinical hyperthyroidism to overt |  |  | 0.04 | 0.82 | 1.30 | 0.67 |
| t26 | Overt hypothyroidism to treated |  |  | 2.24 | 0.99 | 0.48 | 1.80 |
| t36 | Overt hyperthyroidism to treated |  |  | 0.16 | 0.76 | 1.07 | 0.61 |

**Table E. Sensitivity analysis showing the effect of alternative models on the sex- and age-specific prevalence of undiagnosed thyroid disease.**

| Sex | Age group (y) | Observed (%) | Baseline Simulated (%) | Uninformative Priors (%) | Low Variance Priors (%) | Alternative Priors (%) | 85% Adjustment (%) |  |
| --- | --- | --- | --- | --- | --- | --- | --- | --- |
| Subclinical Hypothyroidism | | | | | | | | |
| Male | 20-29 | 3.26 | 1.43 | 1.59 | 1.04 | 1.35 | 1.53 |  |
|  | 30-39 | 3.85 | 2.53 | 2.26 | 1.62 | 2.16 | 2.43 |  |
|  | 40-49 | 3.45 | 2.51 | 1.80 | 2.07 | 2.99 | 2.29 |  |
|  | 50-59 | 4.43 | 2.94 | 2.81 | 2.35 | 3.86 | 3.16 |  |
|  | 60-69 | 5.53 | 4.19 | 4.75 | 3.34 | 4.12 | 4.03 |  |
|  | 70-79 | 6.27 | 5.09 | 2.49 | 4.81 | 5.22 | 5.06 |  |
|  | Mean ^*^ | 4.18 | 2.80 | 2.49 | 2.20 | 2.95 | 2.77 |  |
| Female | 20-29 | 3.38 | 3.24 | 5.71 | 3.43 | 3.78 | 2.53 |  |
|  | 30-39 | 5.15 | 5.32 | 6.98 | 5.17 | 4.78 | 5.00 |  |
|  | 40-49 | 3.76 | 5.39 | 6.77 | 5.47 | 5.02 | 5.59 |  |
|  | 50-59 | 4.37 | 6.12 | 8.04 | 6.88 | 6.38 | 5.94 |  |
|  | 60-69 | 2.59 | 6.14 | 5.92 | 7.43 | 6.99 | 4.78 |  |
|  | 70-79 | 6.44 | 6.81 | 2.84 | 7.36 | 7.01 | 6.45 |  |
|  | Mean ^*^ | 4.14 | 5.26 | 6.39 | 5.61 | 5.34 | 4.85 |  |
| Overt Hypothyroidism | | | | | | | | |
| Male | 20-29 | 0.15 | 0.03 | 0.04 | 0.02 | 0.04 | 0.03 |  |
|  | 30-39 | 0.15 | 0.07 | 0.06 | 0.04 | 0.07 | 0.07 |  |
|  | 40-49 | 0.00 | 0.08 | 0.05 | 0.05 | 0.09 | 0.06 |  |
|  | 50-59 | 0.00 | 0.09 | 0.07 | 0.06 | 0.12 | 0.08 |  |
|  | 60-69 | 0.00 | 0.12 | 0.13 | 0.08 | 0.13 | 0.11 |  |
|  | 70-79 | 0.00 | 0.15 | 0.07 | 0.12 | 0.16 | 0.13 |  |
|  | Mean ^*^ | 0.06 | 0.08 | 0.07 | 0.06 | 0.09 | 0.07 |  |
| Female | 20-29 | 0.00 | 0.06 | 0.16 | 0.07 | 0.10 | 0.06 |  |
|  | 30-39 | 0.17 | 0.16 | 0.18 | 0.13 | 0.15 | 0.13 |  |
|  | 40-49 | 0.00 | 0.17 | 0.19 | 0.15 | 0.15 | 0.16 |  |
|  | 50-59 | 0.24 | 0.19 | 0.21 | 0.18 | 0.19 | 0.16 |  |
|  | 60-69 | 0.29 | 0.20 | 0.17 | 0.21 | 0.22 | 0.14 |  |
|  | 70-79 | 0.00 | 0.21 | 0.09 | 0.21 | 0.22 | 0.16 |  |
|  | Mean ^*^ | 0.11 | 0.16 | 0.18 | 0.15 | 0.16 | 0.13 |  |
| Subclinical Hyperthyroidism | | | | | | | | |
| Male | 20-29 | 0.15 | 0.10 | 0.20 | 0.06 | 0.09 | 0.07 |  |
|  | 30-39 | 0.44 | 0.16 | 0.25 | 0.07 | 0.25 | 0.13 |  |
|  | 40-49 | 0.16 | 0.14 | 0.16 | 0.11 | 0.11 | 0.09 |  |
|  | 50-59 | 0.00 | 0.16 | 0.01 | 0.08 | 0.06 | 0.03 |  |
|  | 60-69 | 0.22 | 0.22 | 0.05 | 0.15 | 0.23 | 0.17 |  |
|  | 70-79 | 0.00 | 0.27 | 0.00 | 0.22 | 0.17 | 0.12 |  |
|  | Mean ^*^ | 0.19 | 0.16 | 0.13 | 0.10 | 0.15 | 0.10 |  |
| Female | 20-29 | 0.19 | 0.62 | 0.27 | 0.51 | 0.48 | 0.56 |  |
|  | 30-39 | 0.66 | 1.05 | 0.43 | 1.17 | 1.25 | 0.91 |  |
|  | 40-49 | 0.54 | 0.82 | 0.26 | 0.55 | 1.19 | 1.28 |  |
|  | 50-59 | 0.00 | 1.07 | 0.09 | 0.22 | 1.07 | 0.70 |  |
|  | 60-69 | 0.29 | 0.61 | 0.03 | 0.50 | 0.48 | 0.69 |  |
|  | 70-79 | 0.00 | 0.56 | 0.00 | 0.71 | 0.14 | 0.54 |  |
|  | Mean ^*^ | 0.34 | 0.82 | 0.23 | 0.64 | 0.87 | 0.83 |  |
| Overt Hyperthyroidism | | | | | | | | |
| Male | 20-29 | 0.15 | 0.03 | 0.15 | 0.02 | 0.02 | 0.02 |  |
|  | 30-39 | 0.00 | 0.04 | 0.09 | 0.02 | 0.05 | 0.04 |  |
|  | 40-49 | 0.00 | 0.04 | 0.06 | 0.03 | 0.04 | 0.03 |  |
|  | 50-59 | 0.00 | 0.04 | 0.04 | 0.03 | 0.02 | 0.01 |  |
|  | 60-69 | 0.22 | 0.06 | 0.02 | 0.04 | 0.05 | 0.04 |  |
|  | 70-79 | 0.00 | 0.08 | 0.01 | 0.06 | 0.05 | 0.05 |  |
|  | Mean ^*^ | 0.06 | 0.05 | 0.07 | 0.03 | 0.04 | 0.03 |  |
| Female | 20-29 | 0.00 | 0.21 | 0.10 | 0.19 | 0.14 | 0.22 |  |
|  | 30-39 | 0.50 | 0.28 | 0.09 | 0.29 | 0.26 | 0.24 |  |
|  | 40-49 | 0.36 | 0.27 | 0.08 | 0.24 | 0.31 | 0.38 |  |
|  | 50-59 | 0.00 | 0.30 | 0.06 | 0.08 | 0.26 | 0.30 |  |
|  | 60-69 | 0.00 | 0.21 | 0.03 | 0.13 | 0.15 | 0.21 |  |
|  | 70-79 | 0.00 | 0.16 | 0.02 | 0.21 | 0.05 | 0.20 |  |
|  | Mean ^*^ | 0.19 | 0.25 | 0.07 | 0.20 | 0.22 | 0.27 |  |

^*^ Mean prevalence weighted by the number of observed individuals in each age group.

**Table F. Sensitivity analysis showing the effect of alternative models on the sex- and age-specific prevalence of diagnosed thyroid disease.** The target (observed) values were from the NHANES, based on whether the participant reported ever being diagnosed with a thyroid condition or if they were taking one of several thyroid replacement or suppression drugs. See methods section and Figure 3 for details. The simulated values are of those ever having been treated for functional thyroid disease. See methods and discussion section for details.

| Sex | Age group (y) | Observed (%) | Baseline Simulated (%) | Uninformative Priors (%) | Low Variance Priors (%) | Alternative Priors (%) | 85% Adjustment (%) |
| --- | --- | --- | --- | --- | --- | --- | --- |
| Male | 20-29 | 0.97 | 0.56 | 0.74 | 0.51 | 0.54 | 0.46 |
|  | 30-39 | 1.56 | 1.71 | 2.24 | 1.64 | 1.73 | 1.58 |
|  | 40-49 | 3.30 | 3.20 | 3.69 | 3.09 | 3.39 | 2.91 |
|  | 50-59 | 4.42 | 4.62 | 5.10 | 4.86 | 5.00 | 4.12 |
|  | 60-69 | 7.63 | 6.45 | 7.65 | 6.90 | 6.94 | 5.78 |
|  | 70-79 | 8.19 | 8.84 | 10.03 | 9.91 | 9.31 | 7.97 |
|  | Mean^+^ | 4.10 | 3.93 | 4.58 | 4.14 | 4.17 | 3.53 |
| Female | 20-29 | 5.04 | 4.45 | 4.76 | 3.36 | 3.73 | 3.42 |
|  | 30-39 | 7.28 | 8.33 | 8.66 | 7.76 | 7.68 | 6.60 |
|  | 40-49 | 13.95 | 13.31 | 13.18 | 13.27 | 13.16 | 11.46 |
|  | 50-59 | 18.07 | 17.66 | 17.36 | 17.63 | 18.14 | 16.22 |
|  | 60-69 | 25.23 | 22.20 | 21.55 | 22.40 | 22.57 | 19.73 |
|  | 70-79 | 28.22 | 25.48 | 23.91 | 27.36 | 25.58 | 22.89 |
|  | Mean^+^ | 15.41 | 14.48 | 14.23 | 14.42 | 14.36 | 12.67 |

^+^ Mean prevalence weighted by the number of observed individuals in each age group.

**References**

1. Flynn RWV, MacDonald TM, Morris AD, Jung RT, Leese GP. The thyroid epidemiology, audit, and research study: thyroid dysfunction in the general population. J Clin Endocrinol Metab. 2004 Aug;89(8):3879–84.

2. Higham NJ, Lin L. On *p*th roots of stochastic matrices. Linear Algebra and its Applications. 2011;435(3):448–463.

3. Caron P, Lehert P, Picard S, Landron F. The DIAGONALE study: a survey designed to analyze the diagnosis and management of goiter in France. Ann Endocrinol (Paris). 2012 Jun;73(3):202–7.

4. Golden SH, Robinson KA, Saldanha I, Anton B, Ladenson PW. Clinical review: Prevalence and incidence of endocrine and metabolic disorders in the United States: a comprehensive review. J Clin Endocrinol Metab. 2009 Jun;94(6):1853–78.

5. Rallison ML, Dobyns BM, Meikle AW, Bishop M, Lyon JL, Stevens W. Natural history of thyroid abnormalities: prevalence, incidence, and regression of thyroid diseases in adolescents and young adults. Am J Med. 1991 Oct;91(4):363–70.

6. Vanderpump MP, Tunbridge WM, French JM, Appleton D, Bates D, Clark F, et al. The incidence of thyroid disorders in the community: a twenty-year follow-up of the Whickham Survey. Clin Endocrinol (Oxf). 1995 Jul;43(1):55–68.
